# Trends in Citations to Books on Epidemiological and Statistical Methods in the Biomedical Literature

**DOI:** 10.1371/journal.pone.0061837

**Published:** 2013-05-07

**Authors:** Miquel Porta, Jan P. Vandenbroucke, John P. A. Ioannidis, Sergio Sanz, Esteve Fernandez, Raj Bhopal, Alfredo Morabia, Cesar Victora, Tomàs Lopez

**Affiliations:** 1 Hospital del Mar Institute of Medical Research (IMIM), Barcelona, CIBER de Epidemiología y Salud Pública (CIBERESP), and School of Medicine, Universitat Autònoma de Barcelona, Catalonia, Spain; 2 Department of Epidemiology, School of Public Health, University of North Carolina at Chapel Hill, Chapel Hill, North Carolina, United State of America; 3 Department of Clinical Epidemiology, Leiden University Medical Center, Leiden, The Netherlands; 4 Department of Hygiene and Epidemiology, University of Ioannina School of Medicine, Ioannina, Greece; 5 Stanford Prevention Research Center, Department of Medicine and Department of Health Research and Policy, Stanford University School of Medicine, and Department of Statistics, Stanford University School of Humanities and Sciences, Stanford, California, United States of America; 6 Cancer Prevention and Control Programme, Institut Català d'Oncologia (ICO-IDIBELL), and School of Medicine, Universitat de Barcelona, L'Hospitalet de Llobregat, Barcelona, Catalonia, Spain; 7 Centre for Population Health Sciences, University of Edinburgh, Edinburgh, Scotland, United Kingdom; 8 Mailman School of Public Health, Columbia University, New York, New York, United States of America; 9 School of Earth and Environmental Sciences and Center for the Biology of Natural Systems, Queens College, City University of New York, Flushing, New York, United States of America; 10 Universidade Federal de Pelotas, Pelotas, Brazil; University of California, Berkeley, United States of America

## Abstract

**Background:**

There are no analyses of citations to books on epidemiological and statistical methods in the biomedical literature. Such analyses may shed light on how concepts and methods changed while biomedical research evolved. Our aim was to analyze the number and time trends of citations received from biomedical articles by books on epidemiological and statistical methods, and related disciplines.

**Methods and Findings:**

The data source was the Web of Science. The study books were published between 1957 and 2010. The first year of publication of the citing articles was 1945. We identified 125 books that received at least 25 citations. Books first published in 1980–1989 had the highest total and median number of citations per year. Nine of the 10 most cited texts focused on statistical methods. Hosmer & Lemeshow's *Applied logistic regression* received the highest number of citations and highest average annual rate. It was followed by books by Fleiss, Armitage, et al., Rothman, et al., and Kalbfleisch and Prentice. Fifth in citations per year was Sackett, et al., *Evidence-based medicine*. The rise of multivariate methods, clinical epidemiology, or nutritional epidemiology was reflected in the citation trends. Educational textbooks, practice-oriented books, books on epidemiological substantive knowledge, and on theory and health policies were much less cited. None of the 25 top-cited books had the theoretical or sociopolitical scope of works by Cochrane, McKeown, Rose, or Morris.

**Conclusions:**

Books were mainly cited to reference methods. Books first published in the 1980s continue to be most influential. Older books on theory and policies were rooted in societal and general medical concerns, while the most modern books are almost purely on methods.

## Introduction

*If one considers the book as the macro unit of thought*

*and the periodical article the micro unit of thought, then…*

Eugene Garfield (1955) [1]

Academic and professional books are both actors and witnesses of their corresponding disciplines. While some books move the frontiers of ignorance, others harvest and synthesize knowledge that seems established [2]–[5]. In principle, analyses of citations to books may shed light on how concepts and methods changed in the course of time whilst a discipline evolved as a field of practice and academic subject; such analyses are also relevant to explore the influence of a discipline on other fields [4]–[8].

Analyses of citations to scientific books are rare and, to our knowledge, there are no comprehensive analyses of citations to books of epidemiology and biostatistics, nor to sets of books in any other of the health and life sciences [6]–[10]. The vast majority of citation analyses involve citations to articles [11]. Studies on citations made by books (to other books or to articles) are also uncommon. In this study we will analyze citations to books made by scientific biomedical articles. Analyzing the bibliometric impact of books is also relevant at a time when the very nature of books and the whole publishing endeavour are experiencing enormous changes.

Analyses of citations to epidemiologic books may complement other analyses on the nature, evolution and endeavours of epidemiology, a particularly integrative science [12], [13]; for decades, epidemiology has been useful to integrate knowledge, methods, reasoning and cultural referents from multiple health and social sciences, including medicine and public health. Hence, uses of epidemiologic and statistical reasoning, knowledge, and methods –foremost, uses in biomedical research– are of broad scientific interest. Indeed, today concepts and methods with strong epidemiological roots and properties seem fruitfully applied ‘within’ and ‘outside’ epidemiology [12]–[15].

As a consequence, there cannot be an exhaustive and fixed list of books on epidemiology and biostatistics. This does not preclude the analysis of an intellectually coherent set of books. Furthermore, since the present study is the first of its kind in epidemiology, a broad perspective is warranted. Therefore, rather than using narrow lists or definitions of what is a book on epidemiology or biostatistics, we will apply a wide, inclusive approach. Specifically, we will aim at including in the study books that have a clear biostatistic or epidemiologic component or dimension, relevant to epidemiology. As we shall see, while all books included are unequivocally on epidemiology or biostatistics, many focus exclusively on biostatistical methods and techniques, and a few others are fundamentally on public health, preventive medicine, clinical epidemiology or other epidemiologic specialties. The Appendix S1 includes a few texts (mostly, essays on health policy and philosophy of medicine) in which epidemiology has a secondary role, but which nevertheless are referents.

The objective of this study was to analyze the number and time trends of citations received from scientific biomedical articles by selected books of epidemiology, biostatistics and related disciplines, including public health and preventive medicine.

## Methods

### Selection of books

Based on academic lists of textbooks [3], [4], [16], books selected by The James Lind Library [17] and the People's Epidemiology Library [18], publishers' catalogues, books cited in other books, and our own teaching and research references, we first searched for citations to over 200 books on epidemiological and statistical methods and concepts. The books initially included were published from 1957 until 2010, and this is the main period of publication covered by the present study; nevertheless, we occasionally expanded the timeframe backwards to assess books published before 1957 that we deemed important texts for reference.

The primary aim was to include books that had a clear biostatistic or epidemiologic component or dimension, relevant to epidemiology and, foremost, to actual uses of epidemiologic methods in research and practice. We accepted books that focus exclusively on biostatistical methods and techniques, but did not consider books on bioinformatics or that focus on mathematical models without emphasising their potential biostatistical application. We also included books on public health, preventive medicine, clinical epidemiology and other epidemiologic specialties. We thus selected books that are pertinent to applied biostatistical and epidemiological methods and techniques; epidemiologic concepts and theory; health services and policies; and substantive epidemiology (e.g., nutritional epidemiology). Books were selected regardless of whether they were aimed at researchers, practitioners, postgraduate students or undergraduates.

If a book received less than 25 citations, it was finally not included in the study; examples of exclusions are given in the Table S1. Books included are listed in the References section in alphabetic order [19]–[304], except if already cited paragraphs [12], [14]. If a book had many editions, only a selection is cited in the References, but we aimed at counting citations to all editions (see Appendix S1).

### Bibliometric analyses

The data source for the study was the Web of Science, produced historically by the Institute for Scientific Information, Inc. (ISI), and in recent years by Thomson – Reuters [305]. The database includes: *a)* the Science Citation Index Expanded (SCI-Expanded), from 1945 to 2011; *b)* the Social Sciences Citation Index, from 1956 to 2011; and *c)* the Arts & Humanities Citation Index, from 1975 to 2011. Thus, while 1945 is the first year of publication of the citing articles, the year of publication of cited books is not restricted by 1945; since articles published from 1945 onwards could cite books published before 1945.

To measure the overall bibliometric impact of each book, we aimed at retrieving all citations received by all editions of the book from the year of publication until 31 December 2011. Thus, our primary measure was the total number of citations received by each book, unadjusted by the number of editions or time since first publication. A second indicator was the average number of citations received by the book per year since publication of the first edition. We also focused on books that received over 1000 citations since publication and books that received an average of more than 40 citations per year since publication. Thus, books published long ago had more time to accumulate citations. Books published in recent decades were *a priori* more likely to receive more than 40 citations per year, since the citing base of articles was larger. Using sampling of papers with random trigrams author searches, we estimated that in the Web of Science the number of papers (and by extrapolation, citations) indexed per year increased approximately 3-fold in the last 3 decades. Finally, we computed an annual citation rate, the number of citations received each year by the selected books at each corresponding time divided by the corresponding number of selected books. To allow for delays in the publication of citing articles, our last search was conducted on February 9, 2012 (always aiming at retrieving citations made by articles published until December 31, 2011). The present study was developed from a minor pilot study conducted in 2006 [16].

Through a ‘cited reference search’, we used combinations of ‘cited author’ and ‘cited work’, the latter referring to the title of a book or to the name of a journal, not to the title of an article. All citations were checked for accuracy as explained in Appendix S1. For each book several possible citation options were searched to allow for different abbreviations and for citation errors. Information on all books was verified against valid information on all editions of the book.

For a given book we included all directly related editions and printings. Citations to each book were searched independently by name of author or editor, and title; all authors or editors (not just the first one) were used in different searches. The data source does not allow to reliably identify chapters of books written by authors other than the book editors [306], unless the title of the book is unique or unambiguous (e.g., *Modern Epidemiology* [250]–[252], *Oxford Textbook of Public Health* [133]–[137]). Thus, if the title was unambiguous, the number of citations includes citations to individual chapters not authored by the book editors; if the title of the book was common, we could not include citations registered with the name of the first author of each chapter (when other than the editors), and some citations to chapters not written by the editors had to be excluded (Appendix S1 and Figure S1). Citing articles were thus identified, and the year of publication of each article was analyzed (through the Web of Science option “analyze results”) to assess time trends in the number of citations to each book.

## Results

### General trends

The 125 books that received 25 citations or more were published between 1913 and 2004 and, therefore, the maximum number of years since publication was 98, and the minimum, 7 (median, 24 years); 100 books (80%) had been published for over 15 years (Table 1). Besides books published in the earlier periods, 46 books (37%) were published in 1980–1989, 35 (28%) in 1990–1999, and 14 (11%) in 2000–2004. We thus see a possible peak of influential books published around 1980–1989; most notably, works by Hosmer & Lemeshow, Rothman et al., Kalbfleisch & Prentice, Breslow & Day, Kleinbaum et al., Rosner, Sackett et al., Schlesselman & Stolley, Hennekens & Buring, Fletcher et al., Bland, Friedman et al., Hulley et al, Feinstein, or Kelsey et al. (Table 2).

**Table 1:** General indicators of the 125 selected books.^a^

Year of 1st. edition	Number of books N (%)		Books that received >1,000 citations N (%)		Books that received >40 citations per year N (%)		Total citations received^b^		Citations per year	
							N	(%)	Mean	Median
1913–1949	3	(2.4)	1	(0.8)	0	(0.0)	3206	1.7	13.4	9.5
1950–1959	2	(1.6)	0	(0.0)	0	(0.0)	1150	0.6	10.8	10.8
1960–1969	6	(4.8)	3	(2.4)	0	(0.0)	8736	4.8	32.0	26.8
1970–1979	19	(15.2)	9	(7.2)	5	(4.0)	50 739	27.7	70.8	12.5
1980–1989	46	(36.8)	19	(15.2)	20	(16.0)	95 555	52.1	80.9	29.3
1990–1999	35	(28.0)	8	(6.4)	10	(8.0)	20 412	11.1	36.0	10.2
2000–2004	14	(11.2)	2	(1.6)	5	(4.0)	5603	3.1	39.1	12.7
Total	125	(100)	42	(33.6)	40	(32.0)	183 401	(100)	57.0	12.5

a All percentages are relative to the 125 books selected for the study because they received at least 25 citations since publication. See also footnotes to Table 2.

b Total number of citations received (at any time) by all books first published in each period.

**Table 2:** Number of citations received by books on epidemiology, biostatistics and related fields.*

Rank^a^	Author(s)^b^	Brief title & references^b^	Edition, year^c^	Years^d^	Citations (total)^e^	Citations per year^e^	
1	Hosmer, Lemeshow	Applied logistic regression [139], [140]	1st., 19892nd., 2000	22	19 276	876.2	
2	Fleiss	Statistical methods for rates and proportions [87]–[89]	1st., 19732nd., 19813rd., 2003	38	17 173	447.4	
3	Armitage, Berry, Matthews	Statistical methods [33]–[36]	1st., 19712nd., 19873rd., 19944th., 2002	40	16 204	405.1	
4	Rothman, Greenland, Lash	Modern epidemiology [250]–[252]	1st., 19862nd., 19983rd., 2008	25	12 256	490.2	
5	Kalbfleisch, Prentice	Statistical analysis of failure time data [155], [156]	1st., 19802nd., 2002	31	8097	261.2	
6	Kleinbaum, Kupper, Muller, Nizam	Applied regression analysis [163]–[166]	1st., 19782nd., 19883rd., 19984th., 2007	33	7662	232.2	
7	Breslow, Day	Statistical methods in cancer research. Vols I & II [59], [60]	1st., 19802nd., 1987	31	6584	212.4	f
8	Kleinbaum, Kupper, Morgenstern	Epidemiologic research [167]	1st., 1982	29	5167	178.2	
9	Rosner	Fundamentals of biostatistics [245]–[249]	1st., 19822nd., 19903rd., 19954th., 20005th., 2006	29	4612	159.0	
10	Streiner, Norman	Health measurement scales [273]–[276]	1st., 19892nd., 19953rd., 20034th., 2008	22	4591	208.7	
11	Sackett, Richardson, Straus, et al.	Evidence-based medicine [258]–[260]	1st., 19972nd., 20003rd., 2005	14	4081	291.5	
12	Sackett, Haynes, Tugwell, Guyatt	Clinical epidemiology [255]–[257]	1st., 19852nd., 19913rd. 2005	26	4069	156.5	
13	Willett	Nutritional epidemiology [302], [303]	1st., 19902nd., 1998	21	3968	189.0	
14	Schlesselman, Stolley	Case-control studies [261]	1st., 1982	29	3512	121.1	
15	McDowell, Newell	Measuring health [190]–[192]	1st., 19872nd., 19963rd., 2006	24	2522	105.1	
16	Pocock	Clinical trials [224], [225]	1st., 1983	28	2431	86.8	
17	Hill	Principles of medical statistics [128]–[130]	^c1^1st., 193710th., 197712th., 1991	74	2123	28.7	
18	Last	Dictionary of epidemiology [12], [172]–[175]	1st., 19832nd., 19883rd., 19954th., 20015th., 2008	28	2112	75.4	
19	Rose et al.	Cardiovascular survey methods [236]–[238]	1st., 19682nd., 19823rd., 2004	43	2072	48.2	
20	Hennekens, Buring	Epidemiology in medicine [127]	1st., 1987	24	2045	85.2	
21	Lilienfeld, Lilienfeld, Stolley	Foundations of epidemiology [176]–[178]	1st., 19762nd., 19803rd., 1994	35	1947	55.6	
22	MacMahon, Pugh, Trichopoulos	Principles and methods [180], [181]	1st., 19602nd., 1996	51	1944	38.1	
23	Pearl	Causality [221]	1st., 2000	11	1913	173.9	
24	Fletcher, Fletcher, Wagner	Clinical epidemiology [90]–[93]	1st., 19822nd., 19883rd., 19964th., 2005	29	1732	59.7	
25	APHA, Emerson, et al.	Communicable diseases [66], [67]	17th., 200019th., 2008	41	1623	39.6	
26	Bland	Introduction medical statistics [56]–[58]	1st., 19872nd., 19953rd., 2000	24	1586	66.1	
27	Friedman, Furberg, DeMets	Fundamentals of clinical trials [100]–[103]	1st., 19812nd., 19853rd., 19964th., 2010	30	1498	49.9	
28	Hulley, Cummings	Clinical research [146], [147]	1st., 19882nd., 2001	23	1475	64.1	
29	Townsend, Davidson	Inequalities in health [293]	1st., 1982	29	1364	47.0	
30	Berkman, Kawachi	Social epidemiology [49]	1st., 2000	11	1311	119.2	
31	Blalock	Causal inference in non-experimental research [52]	1st., 1964	47	1269	27.0	
32	Cochrane	Effectiveness and efficiency [69]	1st., 1972	39	1264	32.4	
33	Clayton, Hills	Statistical models [68]	1st., 1993	18	1260	70.0	
34	Hosmer	Applied survival analysis [141], [142]	1st., 19992nd., 2008	12	1254	104.5	
35	Feinstein	Clinical epidemiology [85]	1st., 1985	26	1217	46.8	
36	Marmot, Wilkinson	Social determinants [186], [187]	1st., 19992nd., 2006	12	1214	101.2	
37	Kelsey, Whittemore, Evans, Thompson	Methods in observational epidemiology [157], [158]	1st., 19862nd., 1996	45	1196	26.6	
38	Petitti	Meta-analysis [222], [223]	1st., 19942nd., 2000	17	1125	66.2	
39	McKeown	The role of medicine [193]	1st., 1976	35	1101	31.5	
40	Rose	The strategy of preventive medicine [233]	1st., 1992	19	1066	56.1	
41	Schottenfeld, Fraumeni	Cancer epidemiology [262]–[265]	1st., 19751st., 19822nd., 19963rd., 2006	36	1055	29.3	
42	Kleinbaum, Klein	Logistic regression [168], [169]	1st., 19942nd., 2002	17	1001	58.9	
43	Holland, Detels, Knox, et al.	Oxford textbook of public health [132]–[137]	1st., 19842nd., 19913rd., 19974th., 20025th., 2009	27	977	36.2	
44	Rosenau, Maxcy, Sartwell, Last, et al.	Preventive medicine [236]–[243]	1st., 19137th., 195110th., 197311th., 198013th., 1992	98	931	9.5	g
45	Doll, Peto	Causes of cancer [73]	1st., 1981	30	912	30.4	h
46	Kahn, Sempos	Statistical methods in epidemiology [154]	1st., 1989	22	901	41.0	
47	Sudman, Bradburn, Wansink	Asking questions [281], [282]	1st., 19822nd., 2004	29	795	27.4	
48	Miettinen	Theoretical epidemiology [195]	1st., 1985	26	794	30.5	
49	Kleinbaum	Survival analysis [170], [171]	1st., 19972nd., 2005	15	783	52.2	
50	Mausner, Bahn	Epidemiology [188], [189]	1st., 19742nd., 1985	37	767	20.7	
51	Meinert	Clinical trials [194]	1st., 1986	25	702	28.1	
52	Patrick, Erickson	Health status and health policy [220]	1st., 1993	18	680	37.8	
53	Feinstein	Clinimetrics [86]	1st., 1987	24	650	27.1	
54	Szklo, Nieto	Epidemiology. Beyond the basics [286]	1st., 2000	11	642	58.4	
55	Gordis	Epidemiology [108]–[110]	1st., 19962nd., 20003rd., 2004	15	641	42.7	
56	Checkoway, Pearce, Kriebel	Occupational epidemiology [64], [65]	1st., 19892nd, 2004	22	619	28.1	
57	Rothman	Epidemiology: an introduction [254]	1st., 2002	9	599	66.6	
58	Rosen	A history of public health [234], [235]	1st., 19582nd., 1993	53	595	11.2	
59	Morris	Uses of epidemiology [199]–[201]	1st., 19572nd., 19643rd., 1975	54	555	10.3	
60	Khoury, Beaty, Cohen	Fundamentals of genetic epidemiology [159]	1st., 1993	18	533	29.6	
61	Susser	Causal thinking [283]	1st., 1973	38	475	12.5	
62	Adami, Hunter, Trichopoulos	Textbook of cancer epidemiology [28], [29]	1st., 20022nd., 2008	9	440	48.9	
63	Blalock	Causal models in social sciences [53], [54]	1st., 19712nd., 1985	40	436	10.9	
64	Morrison	Screening [202], [203]	1st., 19852nd., 1992	26	353	13.6	
65	Margetts, Nelson	Nutritional epidemiology [182], [183]	1st., 19912nd., 1997	20	325	16.3	
66	Monson	Occupational epidemiology [196], [197]	1st., 19802nd., 1990	31	297	9.6	
67	Bailar, Mosteller, Hoaglin	Medical uses of statistics [40]–[42]	1st., 19862nd., 19923rd., 2009	25	293	11.7	
68	Abramson	Survey methods [19]–[24]	1st., 19742nd., 19793rd., 19844th., 19905th., 19996th., 2008	37	289	7.8	
69	Strom	Pharmacoepidemiology [277]–[280]	1st., 19892nd., 19943rd., 20004th., 2005	22	264	12.0	
70	Elwood	Causal relationships in medicine [78]–[80]	1st., 19882nd., 19983rd., 2007	23	263	11.4	
71	Kahn	Introduction to epidemiologic methods [153]	1st., 1983	28	262	8.6	
72	Gore, Altman	Statistics in practice [111]	1st., 1982	29	249	8.6	
73	Friedman	Primer of epidemiology [95]–[99]	1st., 19742nd., 19803rd., 19874th., 19945th., 1998	37	226	6.1	
74	Jensen, et al.	Cancer registration [152]	1st., 1991	20	208	10.4	
75	Schulte, Perera	Molecular epidemiology [266]	1st., 1993	18	204	11.3	
76	Beaglehole, Bonita, Kjellström	Basic epidemiology [48]	1st., 19932nd., 2006	18	190	10.6	
77	Robertson	Injury epidemiology [230]–[232]	1st., 19922nd., 19983rd., 2007	19	184	9.7	
78	Hulka, Wilcosky, Griffith	Biological markers in epidemiology [144]	1st., 1990	21	184	8.8	
79	Teutsch, Churchill	Principles and practice of public health surveillance [287], [288]	1st., 19942nd., 2001	17	173	10.2	
81	Khoury, Little, Burke	Human genome epidemiology [161], [162]	1st., 20032nd., 2010	8	171	21.4	
81	Jekel, Elmore, Katz	Epidemiology, biostatistics and preventive medicine [148]–[150]	1st., 19962nd., 20013rd., 2007	15	163	10.9	
82	Murphy	The logic of medicine [210], [211]	1st., 19762nd., 1997	35	152	4.3	
83	Greenwood	Epidemics and crowd diseases [118]	1st., 1935	76	152	2.0	
84	Elliott, et al.	Geographical and environmental epidemiology [74]	1st., 1992	19	151	7.9	
85	Greenberg, et al.	Medical epidemiology [113]–[116]	1st., 19932nd., 19963rd., 20014th., 2005	18	147	8.2	
86	Polgar, Thomas	Introduction to research in the health sciences [224]	1st., 1988	23	139	6.0	
87	Elston, Johnson	Essentials of biostatistics [75], [76]	1st., 19872nd., 1994	24	134	5.6	
88	Hill	Statistical methods [131]	1st., 1962	49	131	2.7	
89	Marmot, Elliott	Coronary heart disease epidemiology [184], [185]	1st,. 19922nd., 2005	19	128	6.7	
90	Barker, Rose	Epidemiology in medical practice [43]–[47]	1st., 19762nd., 19793rd., 19844th., 19905th., 1998	35	128	3.7	
91	Fox, Hall, Elveback	Epidemiology [94]	1st., 1970	41	125	3.0	
92	Doll	Prevention of cancer [71], [72]	1st., 19672nd., 2008	44	124	2.8	
93	Friis, Sellers	Epidemiology for public health practice [104]–[107]	1st., 19962nd., 19993rd., 20044th., 2009	15	115	7.7	
94	Brownson, Baker, Leet, Gillespie	Evidence-based public health [61]	1st., 2003	8	114	14.3	
95	Nelson, Williams, Graham	Infectious diseases epidemiology [212], [213]	1st., 20012nd., 2006	10	111	11.1	
96	Weiss	Clinical epidemiology [297], [298]	1st., 19862nd., 1996	25	111	4.4	
97	Rothman	Causal inference [253]	1st., 1988	23	107	4.7	
98	Hartzema, Porta, Tilson, Chan	Pharmacoepidemiology [123]–[126]	1st., 19882nd., 19913rd., 19984th., 2008	23	106	4.6	
99	Linet	The leukemias [179]	1st., 1985	26	105	4.0	
100	Ahlbom, Norell	Introduction to modern epidemiology [30], [31]	1st., 19842nd., 1990	27	97	3.6	
101	Coggon, Rose, Barker	Epidemiology for the uninitiated [70]	1st., 1993	18	92	5.1	
102	Holland	Screening [138]	1st., 1990	21	87	4.1	
103	Gregg	Field epidemiology [119]–[121]	1st., 19962nd., 20023rd., 2008	15	85	5.7	
104	Vineis, et al.	Metabolic polymorphisms [295]	1st., 1999	12	83	6.9	
105	Abramson, Abramson	Making sense of data [25]–[27]	1st., 19882nd., 19943rd., 2001	23	83	3.6	
106	Patrick, Peach	Disablement [219]	1st., 1989	22	72	3.3	
107	Khoury, Burke, Thomson	Genetics and public health [160]	1st., 2000	11	69	6.3	
108	Aschengrau, Seage	Essentials of epidemiology [37], [38]	1st., 20032nd., 2008	8	67	8.4	
109	Elston, Olson, Palmer	Biostatistical genetics [77]	1st., 2002	9	65	7.2	
110	Starfield, et al.	Effectiveness of medical care [270]	1st., 1985	26	60	2.3	
111	Toniolo, et al.	Biomarkers in cancer epidemiology [292]	1st., 1997	14	58	4.1	
112	Morton, Hebel, McCarter	A study guide to epidemiology and biostatistics [204]–[209]	1st., 19792nd., 19843rd., 19904th., 19965th., 20016th., 2005	32	57	1.8	
113	Timmreck	Introduction to epidemiology [289]–[291]	1st., 19942nd., 19983rd., 2002	17	55	3.2	
114	Silman, MacFarlane	Epidemiological studies [268], [269]	1st., 19952nd., 2002	16	48	3.0	
115	Bhopal	Concepts of epidemiology [50], [51]	1st., 20022nd., 2008	9	43	4.8	
116	Jenicek	Epidemiology [151]	1st., 1995	16	40	2.5	
117	Greenland	Evolution of epidemiologic ideas [117]	1st., 1987	24	34	1.4	
118	Olsen, Trichopoulos, Saracci	Teaching epidemiology [215]–[217]	1st., 19922nd., 20013rd., 2010	19	32	1.7	
119	Morabia	History of epidemiologic methods [14]	1st., 2004	7	31	4.4	
120	Buck, et al.	Readings [62]	1st., 1988	23	30	1.3	
121	Farmer, Miller, Lawrenson	Lecture notes [81]–[84]	1st., 19772nd., 19833rd., 19914th., 1996	34	30	0.9	
122	Holford	Multivariate methods in epidemiology [132]	1st., 2002	9	27	3.0	
123	Armenian, Shapiro	Epidemiology and health services [32]	1st., 1998	13	27	2.1	
124	Stolley, Laskey	Investigating disease patterns [272]	1st., 1995	16	27	1.7	
125	White, Henderson	Epidemiology as a fundamental science [299], [300]	1st., 19762nd., 1980	35	25	0.7	

* Based on ISI - Thomson - Reuters Web of Science [305].

a Ranked by total number of citations received by all editions in all years (from year of publication until 31 December 2011).

b For identification purposes: only the first authors and an abbreviated title are mentioned; the full reference is provided in the list of references.

c All editions, except for Hill's *Principles of medical statistics*.^c1^

d Years elapsed from first edition until the end of 2011.

e Simple average from year of publication until 31 December 2011 (all editions).

f The data source does not allow to distinguish citations to each volume.

g While only the main editions are mentioned in the Table and References, citations to all editions were counted.

h The corresponding (identical) journal article [337] received 2369 citations.

All 10 top-cited books had an edition (not necessarily the first) during 1980–1989. This decade also stands out when we analyze the relative proportion of the study books that received over 1000 citations: such proportion peaked at near 15% in 1980–1989, as did the relative proportion of texts that received more than 40 citations per year since publication (Table 1).

**Figure 1:**
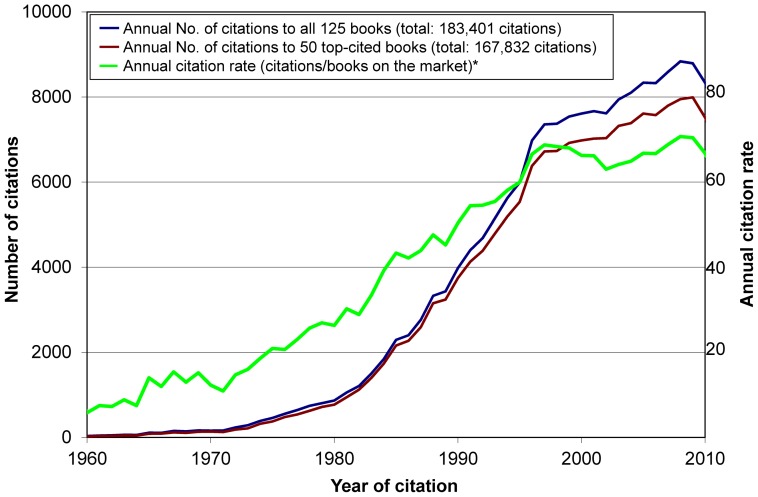
Total number of citations received by books included in the study each calendar year, and annual citation rate.* [ Footnote: *Annual citation rate: Number of citations received each year by the 122 books available in the market at each corresponding time divided by the number of the 122 books in the market at each corresponding time. ]

The total number of citations received by the 125 books was 183 401, of which 52% were to books first published in 1980–1989. Books first published in this decade also had the highest mean and median number of citations per year since publication (Table 1). The crude number of citations to the selected books increased substantially from 1990 to the mid-1990s, and remained stable thereafter: there were 160 citations in 1970, 869 citations in 1980, 3980 citations in 1990, 7356 citations in 1997, and 8838 in 2008 (Figure 1); in such years the annual citation rate was 12.3, 26.3, 50.4, 68.7 and 70.7 citations/book, respectively.

Among the 50 top-cited books, 15 are completely or strongly on applied biostatistical methods, and the contents of at least a further three is heavily on quantitative methods (18 out of 50 = 36%). At least 33 of such 50 books (two-thirds) have strong methodological contents.

Nine of the top 10 books are general works on statistical or epidemiological methods: 7 of 10, including the top three, are strongly focused on statistics, while two address epidemiological methods as well (4th. and 8th. places, also with strong statistical contents). Ranking 10th. and 15th., we find texts on health measurement (Table 2).

### Influential individual titles

Hosmer & Lemeshow's *Applied logistic regression* [139] is the title that received the highest number of citations and the highest average annual rate since publication. It is followed by Fleiss [87], Armitage et al. [33], Rothman et al. [250], and Kalbfleisch & Prentice [155] (Table 2). None of the previous 5 texts is affected by limitations on retrieving citations to book chapters not authored by the book editors (see Methods). Except for Hosmer & Lemeshow's, whose ranking does not change, the ranking of all other 9 books among the top 10 by total citations changes slightly when we use the average number of citations per year: ranking 2nd. is then Rothman et al., 3rd. is Fleiss, and 4th. is Armitage et al. Fifth in citations per year is Sackett et al. *Evidence-based medicine*. Of books that have been on the market for less than 15 years, only 9 have had more than 40 citations per year: as just mentioned, Sackett et al. *Evidence-based medicine* (ranking 11th. by total citations and 5th. by citations/year), Pearl (23rd. and 12th., respectively), Berkman & Kawachi (30th. and 16th.), Hosmer's *Survival analysis* (34th. and 18th.), Marmot & Wilkinson (36th. and 19th.), Rothman's *Introduction* (57th. and 25th.), Szklo & Nieto (54th. and 31st.), Kleinbaum's *Survival analysis* (49th. and 34th.), and Adami et al. (62nd. and 36th.).

**Figure 2:**
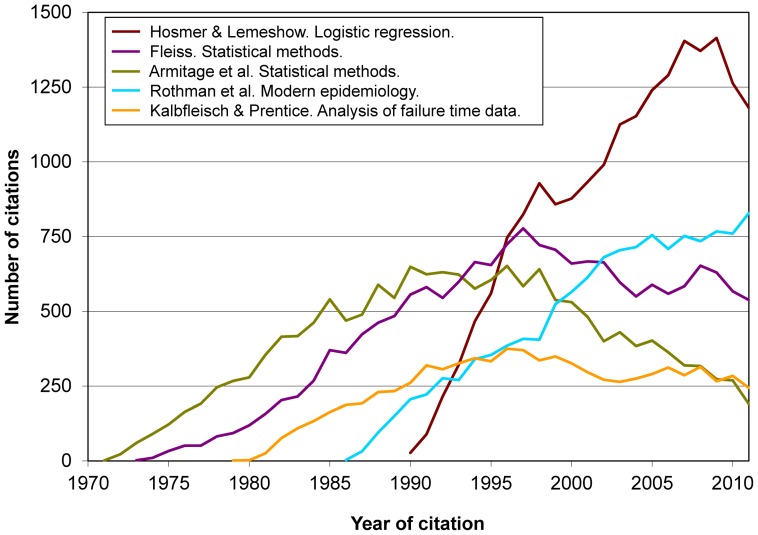
Evolution over time of citations received by the study 5 top-cited books (total, 73,006 citations).

**Figure 3:**
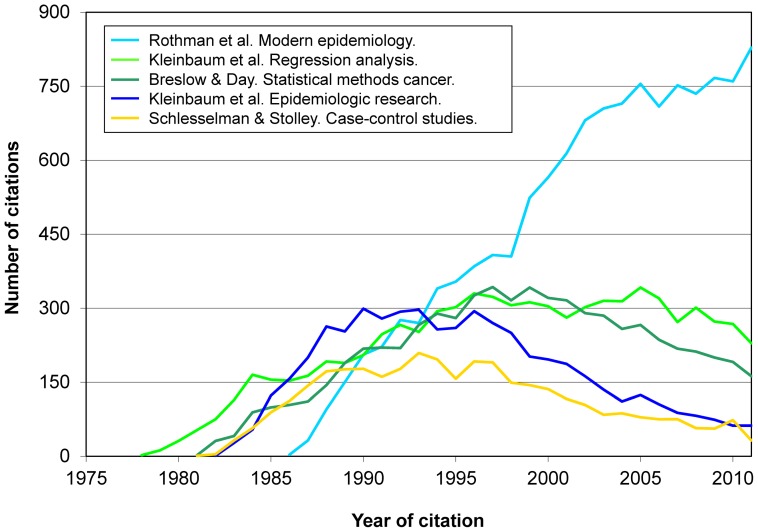
Evolution over time of citations received by 5 books on epidemiological and statistical methods (total, 35,181 citations).

Figure 2 shows that Hosmer & Lemeshow clearly became the annually most cited book from the mid 1990s on. *Modern epidemiology* also received a large number of citations, at a rate that nowadays remains upward, in contrast with the three other books shown in Figure 2. This view is complemented by Figure 3, where we see the evolution of citations received by *Modern epidemiology* and 4 other books with similar and different features. In such Figures there is no presence of books of the so-called “first and second generations” [2] (e.g., by Greenwood, Hill, Morris, MacMahon & Pugh, Susser, Lilienfeld & Lilienfeld).

**Figure 4:**
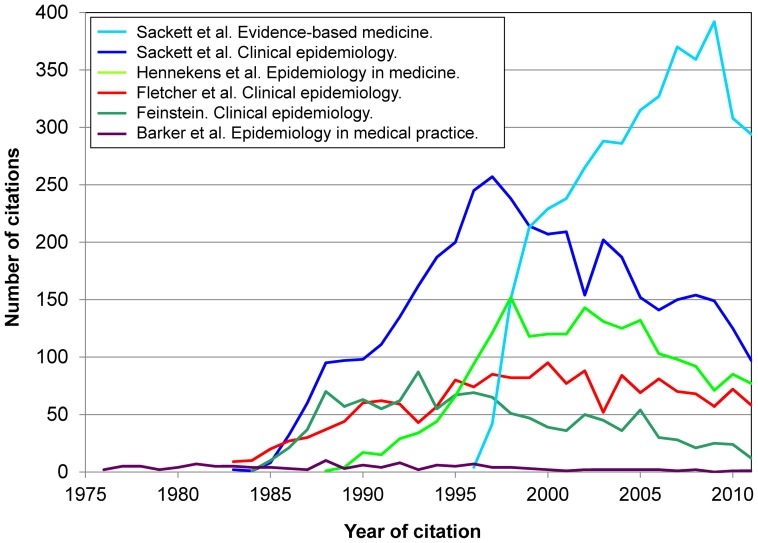
Citations to 6 books on clinical epidemiology (total, 13,272 citations).

Texts on clinical epidemiology and evidence-based medicine were in positions 11th., 12th., 20th., 24th., 28th or 35th. (by total citations) (Table 2). While other texts on clinical epidemiology appeared earlier on, they were seldom cited. The bloom of clinical epidemiology –and later, evidence-based medicine– in the 1980s is apparent in Figure 4; the Figure suggests that the academic influence of clinical epidemiology was largely due to authors from North America. It should be noted that the third edition of Sackett et al. *Clinical epidemiology* [255]–[257] is actually a different book with different contents from the previous two editions, whereas all editions of Fletcher et al. *Clinical epidemiology* [90]–[93] are updated revisions of essentially the same text.

**Figure 5:**
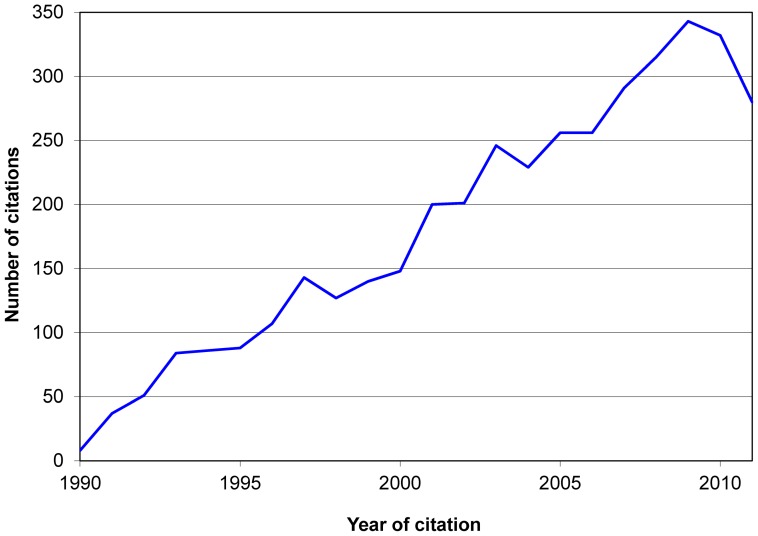
Evolution of the 3,968 citations received by W. Willett's ‘Nutritional epidemiology’.

Willett's book on nutritional epidemiology, also strongly focused on methods, ranked 13th.; it is the first book on that specialty. Figure 5 likely reflects an important part of the surfacing of nutritional epidemiology.

**Figure 6:**
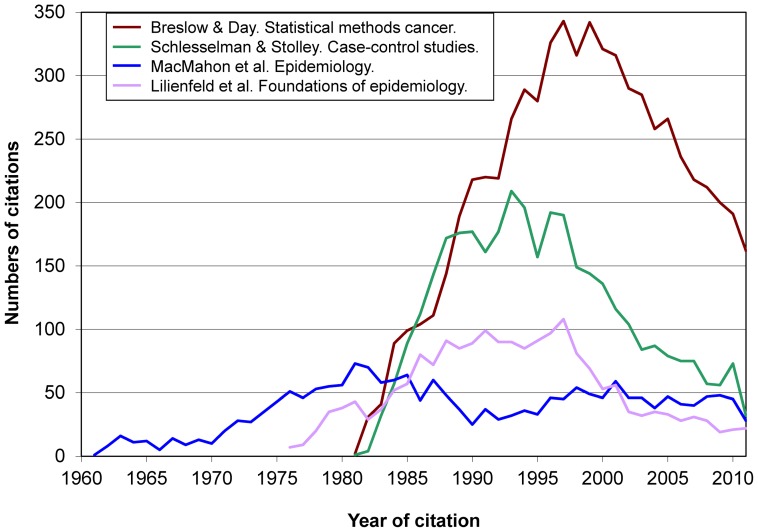
Citations to 2 books preferentially used in research and to 2 books preferentially used in teaching (total, 13,987 citations).

Besides introductory works on clinical epidemiology, introductory textbooks on epidemiology do not appear until positions 21st. (Lilienfeld & Lilienfeld) and 22nd. (MacMahon et al., “the first formal epidemiologic textbook ever published in the United States” [5]); the two texts have remarkably similar figures (Table 2). Figure 6 deliberately contrasts books of the “second generation” [2] (MacMahon et al., Lilienfeld & Lilienfeld) (which we deem highly influential in the education of many epidemiologists and other health professionals, but rarely considered appropriate as a supporting citation in a scientific article) and of the “third generation” (Breslow & Day, and Schlesselman & Stolley) (commonly used in teaching but also acceptable as a citation in a scientific article). They are followed by what we may call the ‘fourth generation’, i.e., texts by Kleinbaum et al., Rothman et al., or Szklo & Nieto. Figure 6 reflects the higher weight in the citing literature of research methods-oriented books over textbooks primarily used in the classroom.

**Figure 7:**
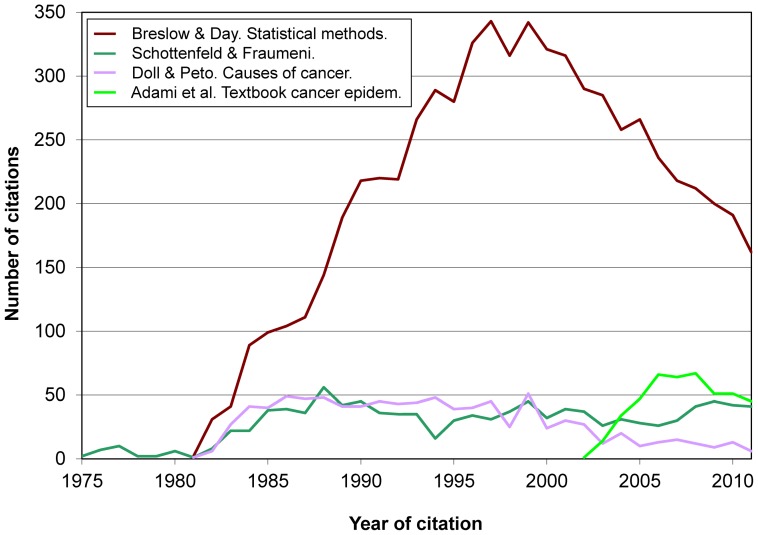
Citations to 5 books on cancer epidemiology (total, 8,991 citations).

Results in Table 2 permit to assess the evolution of citations received by different kinds of books on a similar subject. For instance, Figure 7 shows such evolution for the book by Breslow & Day (a text in two volumes which, though sponsored by the International Agency for Research on Cancer, is more centered on methods than on cancer control), Schottenfeld & Fraumeni *Cancer epidemiology* [262]–[265] (a classic example of a large reference volume synthesizing substantive knowledge on cancer, with relatively less attention to methods), Doll & Peto *Causes of cancer* [73] (a highly influential review, commissioned by the US Congress) [307], and Adami et al. *Textbook of cancer epidemiology* [28], [29] (an emerging textbook summarizing substantive knowledge in a smaller format).

Other general introductory texts ranked 26th. (Bland), 50th. (Mausner & Bahn), 55th. (Gordis), 57th. (Rothman), 71st. (Kahn), 72nd. (Gore & Altman), 73rd. (Friedman) or 76th. (Beaglehole et al.). The text by Ahlbom & Norell (100th.) is also an example of a book used in introductory courses and not aiming to be highly cited in the scientific literature. The same applies to Coggon et al. (101st.) [70], a book stemming from a series of articles in a medical journal; also originating in series of articles published in medical journals are books by Hill [128], Bailar & Mosteller [40], and –already mentioned– Gore & Altman [111].

While many of the books included in the study discuss causal inference, books explicitly centered on causality are in positions 23rd. (Pearl), 31st. and 63rd. (Blalock), 61st. (Susser) (“the first book-length treatment of causal reasoning in epidemiology” [308]), and 70th. (Elwood) (Figures S2 and S3). Besides some books on clinical epidemiology, the first book clearly addressed to public health and clinical practice is the American Public Health Association (APHA) *Control of communicable diseases manual* (ranking 25th.). There were three books devoted solely to clinical trials (in positions 16th., 27th. and 51st.) (Table 2, and Figure S4). The top ranking books on social epidemiology were in positions 29th., 30th. and 36th. (time trends are shown in Figure S5).

**Figure 8:**
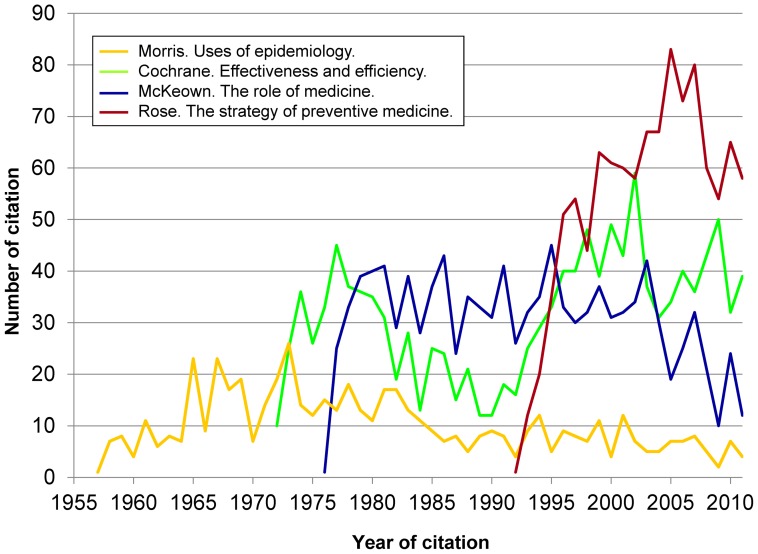
Citations to 4 books on concepts, theory and policies (total, 3,986 citations).

None of the 25 top-cited books has the theoretical, conceptual, historical or sociopolitical approach of works by Cochrane (32nd.) [69], McKeown (39th.) [193], Rose (40th.) [233], Morris (59th.) [199], or Murphy (82nd.) [210] (Figure 8). Figures for the books by Cochrane, McKeown, and Rose (about 1000 total citations each, and 30–60 citations/year) are similar to figures for popular texts outside epidemiology by Kleinman (*The illness narratives*) [309], Fuchs (*Who shall live?*) [310], or Knowles (*Doing better and feeling worse*) [311] (Appendix S1, section 2).

The most cited books on historical aspects of epidemiology and public health were Rosen's (58th., after 53 years since first publication; average of 11 citations/year) [234] and Morabia's (119th. after 7 years; 4 citations/year) [14]. Some of the extensive textbooks (e.g., on cancer epidemiology [262]–[265], public health [133]–[137], and preventive medicine [236]–[244]) were in positions 41st., 43rd. and 44th., respectively. Their figures are particularly affected by current limitations on retrieving citations to book chapters not authored by book editors. Other texts on substantive specialties were in positions 56th. and 66th. (occupational epidemiology), 60th. (genetic epidemiology), 62nd. (cancer epidemiology), 64th. and 102nd. (screening), 65th. (nutritional epidemiology), 69th. (pharmacoepidemiology) or 77th. (injury epidemiology). Citations to the text on cardiovascular survey methods by Rose et al. [236] were estimated to be 2072 (ranking 19th.), and to the book on coronary heart disease by Marmot and Elliott [184], 128 (89th). Six texts on molecular and genetic epidemiology ranked 75th. (Schulte & Perera), 78th. (Hulka et al.), 80th. and 107th. (Khoury et al.), 104th. (Vineis et al.), 109th. (Elston et al.), and 111th. (Toniolo et al.). The only book on meta-analysis in Table 2 is Pettiti's (38th., with over 1100 citations).

### Other analyses and comparisons

**Figure 9:**
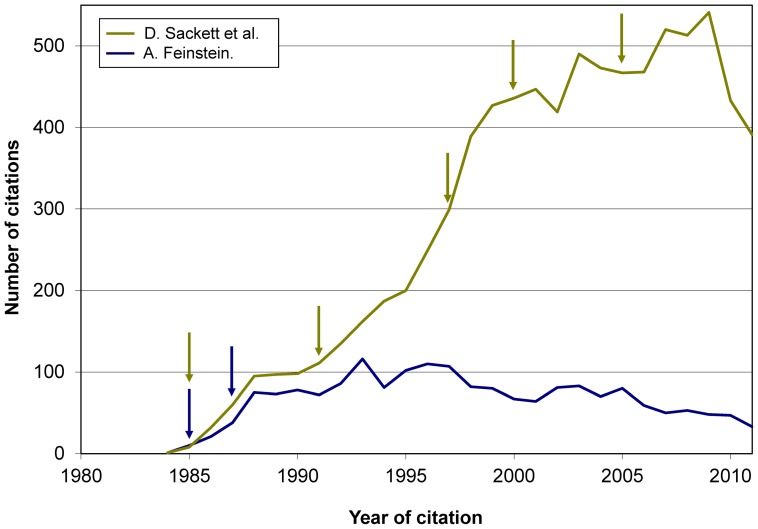
Citations to 2 books by D. Sackett et al. (8,150 citations) and 2 books by A. Feinstein (1,867 citations). [ Footnote: Arrows show the year of publication of all editions of each book. ]

**Figure 10:**
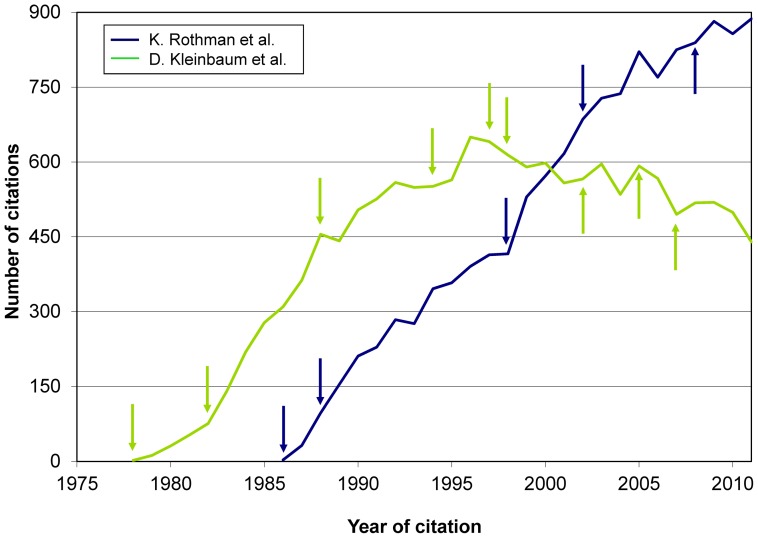
Citations to 3 books by K. Rothman et al. (12,962 citations) and 4 books by D. Kleinbaum et al. (14,613 citations). [Footnote: Arrows show the year of publication of all editions of each book. ]

The study aims did not include to assess the scholarly performance or influence of an author. It will hence suffice to illustrate how citations to books may complement other analyses on such performance. For instance, Figure S5 may add to existing views on the influence of the two books by H. Blalock selected for the study: citations peaked in the 1970s, and were still significant in subsequent decades. Another example: *a priori* both A. Feinstein and D. Sackett had a strong influence on clinical epidemiology and the methodology of clinical research; yet, a complementary perspective is gained when looking at the citations during the past 25 years to the 2 most cited books by Feinstein (1867 citations) and 2 books by Sackett et al. (8148 citations) (Figure 9). The evolution over time of the bibliographic impact of some books is also illustrated by the two graphs contrasting trends in citations received by 3 books by K. Rothman et al. (12 962 citations) and 4 books by D. Kleinbaum et al. (14 613 citations) (Figure 10). Other examples are given in Figures S6, S7, S8, S9, S10.

## Discussion

The most prominent pattern in our findings was that the books were mainly cited to reference methods and statistical techniques. The number of citations was larger for books that provide unique sources for (general or specific) methods used in biomedical research papers than for books that innovated in theory, discuss concepts or are preferentially used in teaching. Nine of the 10 most cited texts focused on widely used statistical methods. Some books that have likely been influential –scientifically, theoretically, educationally– in biomedical research or health policy were not highly cited; notable examples include texts by Hill [128], Susser [283], Rose [233], Morris [199], Cochrane [69], or McKeown [193]. Their lower citation figures may partly be due to earlier publication, when all works received less citations because a lower number of articles was published than later on. For instance, Hill's books ranked 17th. [128]–[130] and 88th. [131] (Table 2); the first book [128]–[130] was first published in 1937, decades ahead of all texts in the first 40 positions (Figure S6). Older books on theory and policies were rooted in societal and general medical concerns, while the most modern books are almost purely on methods, particularly statistical methods.

By study design, 1945 was the first year of publication of the citing articles; yet, plenty of books on statistics and epidemiology existed before 1945: several books by Major Greenwood (1880–1949) (e.g., his major opus, *Epidemics and Crowd Diseases*) [118]; Woods and Russels book on teaching statistics, which preceded A.B. Hill's; the *Epidemiologic Essays* by Crookshank from 1930; *Epidemiology Old and New*, by Hamer, from 1928; or *The Natural History of Disease*, by Ryle, of 1948, which inspired countless public health professionals [14], [15]. So, while we had to stick to a relatively limited time-frame we could also make some comparisons with a few selected classics, including the 3 books published between 1913 and 1949 (Table 1) [118], [128], [236].

The lower citation figures of books on concepts and policies than of books on methods do not necessarily mean that the citing literature pays less attention to theoretical than to methodological issues: such figures may reflect that authors of articles tend to take basic ideas and concepts for granted, as part of the core or background knowledge of most readers, and therefore not needing to be referenced [2], [4], [8], [13]. Citation frequency is only one and modest proxy for influence [1]. A main reason why books on biostatistics top the list may be that they provide convenient citations for statistical techniques widely used in biomedical research. Most biomedical journals request such citations. An important issue is also that findings must be replicable and, therefore, authors cite methods in detail.

It was beyond the scope of the study to register: *a)* whether citations were to a single technique explained in a specific part of the cited book or a generic citation; *b)* who was citing (e.g., what epidemiologic or clinical specialties, substantive areas, academic groups); or *c)* the appropriateness of the citation. As citations to articles [1], [13], [312]–[315], citations to books may be shaped by convenience and habit rather than by a genuine need for scientific reference. Many citations to articles are non-specific, not-justified, or wrong [316]. We are not aware of similar studies on book citations, but we suspect that these problems may also be common with citations to books.

The total citing base (over 20 millions of articles with over half a billion citations) [317]–[319] certainly includes many more empirical studies (which require methodological and technical citations) than articles on theoretical and conceptual issues. However, a key question is whether current practices of referencing do justice to the needs of science. For instance, logistic regression has many variants and implementations; it is important that a research paper explains exactly what was done in the model selection, building, analysis, and validation in the specific application. If instead the paper provides no information on these issues, but just a citation to a book, then science is not well served. Future studies could assess how often the citation also included reference to specific pages or chapters in the book, and whether the citation was specific or just to the whole book.

Future research could use a random sample of the citing articles, their citations to the relevant books, and the 3 factors mentioned above; e.g., see the appropriateness and specificity of the citations, and whether they act as an alibi for not mentioning important methodological details. Such study will need retrieving and data extracting the articles; so, it will have to be limited to a few hundred references, to examine the sentence and context where the reference appears. A future study could also identify influential books that we may have overlooked, use other reliable data sources to measure citations received by books, or obtain data on annual book sales, which could be compared to citation data.

Books primarily meant for students, some of which have likely been highly sold and effective in classrooms –and most influential in coalescing epidemiologic thinking– collected relatively less citations. Important examples include books by Barker et al. [43], MacMahon et al. [180], the Lilienfelds [176], Gordis [108], or Szklo & Nieto [286]. Being used in research is not the primary purpose of such textbooks [2], [3]. Knowledge sifted into a textbook has become normal, background, foundational knowledge [4]; there is no need to reference the material, even though it may be of great importance. A rather different case is a book not just addressed to students, but also to practicing physicians: Sackett's et al. *Evidence-based medicine* [258], [259], which gathered more than 4000 citations. An analysis of the development of professional and scientific movements as evidence-based medicine could use and expand some of the study findings [12], [14], [320], [321]. It would in turn provide new insights into the actual influence of some books; e.g., Cochrane's [69] and Sackett's [258], [259] roles in the creation of the Cochrane Collaboration and the UK National Institute for Health and Clinical Excellence (NICE) [8].

Analyzing citations received by books of epidemiology published over the last 50 years is also a way of tracing the history of the discipline and, to a lesser extent, of biomedical research. Citations contribute to analyze the evolution of the *corpus* of methods and concepts used by epidemiology and by the disciplines with which epidemiology interacts [2], [8], [12]–[15], [322], [323]. The rise of multivariate methods is a relevant example; it clearly explains a large portion of the citations made by papers published in the 1980s. Such methods were then not yet ‘normal science’ [4] in epidemiological and clinical research, and were hence in need of referencing.

The decade 1980–1989 stood out as the period in which more highly-cited books were published. Although we ignore the total number of books on epidemiology and biostatistics published in each period, missing books are unlikely to be that influential so as to change this finding. Overall, other than a few dozen highly-cited books, most books had limited or minimal citation impact. Books published in recent years do not seem to have the influence of textbooks published for the first time in the 1980s. Moreover, the total annual citations to books have been rather steady in the last 15 years, or even declining for many specific texts. Given the large increase in the annual number of citations registered in the Web of Science, the relative citation influence of the study books is probably decreasing sharply, when seen as a proportion of total citations. The total number of citations to the study books (some 183 000 over half a century) probably represents a small fraction of the total citations to the epidemiological and statistical literature, which we estimate to be several million among the over half a billion citations indexed by the Web of Science. Even the few highly-cited books seem to have lost their relative impact in the last 2 decades, because between 1980 and 2011 the number of citing articles (and, correspondingly, citations) increased several-fold. It would next be necessary to perform some adjustment of the citation counts for the total number of citations each year. A comparison with books from other disciplines would then also be meaningful. In spite of its limitations (e.g., choice of citing journals, focus on research-oriented, largely Anglo-Saxon journals) the study data source has long enabled relevant analyses [1], [6], [312]–[315], [324].

We are not aware of any typology of books that could be useful for the present study. Although it would have been possible to use narrower definitions of what is a book on epidemiology and biostatistics, we chose a wide and inclusive approach, which yields a broad perspective and enables a richer set of comparisons; it also allows readers to conduct other, more focused comparisons. The inclusive approach seems particularly appropriate at a time when no other analyses are yet available.

In spite of the study limitations, we think that just comparing the number and trends of citations to the books that we identified is of interest. The uses of books and their true impact on research and professional practices are questions that often seem to escape analysis. Yet books comprise an important part of scientific production, and evaluations of such production would benefit from considering books along scientific articles. Several projects are under development to register citations to books [11], [325], [326]. However, none has at present the scope in time and the qualitative features of the present study. Analyses of citations to books are just one approach to analyses of uses of books. An additional task for future research would be to analyze the relative importance of books and articles in the exercise of scientific leadership and social influence by selected authors. An example is shown in Figure S10, which shows that the increase in the number of citations to the book on logistic regression by Hosmer & Lemeshow did not come at the expense of the highly cited article by Mantel & Haenszel [327].

Several influential authors wrote more than one or two books. In the case of M. Susser, for instance, his *Sociology in medicine* [328] has accumulated over 300 citations. To value Susser's oeuvre it is thus necessary to assess several other books of his [283], [308], [329]–[331]. J. Last, D. Sackett, O. Miettinen, J. Morris, W. Holland, D. Kleinbaum, A. Feinstein, K. Rothman, M. Khoury, H. Blalock, G. Friedman or J. Abramson are other –very different, obviously– examples of authors with more than one influential book [3], [4], [5], [14], [15], [323], [332], [333]. Some books on research methods were less cited than articles on the same methods; for instance, some papers by Hill [334] or Miettinen [335] have each accumulated over 2400 and 1500 citations, respectively [6]. However, seminal papers published long ago received comparatively few citations; for instance, a paper by Jerome Cornfield [336], published over 60 years ago, received less than 450 citations. The review on the causes of cancer by Doll & Peto [307] provides a telling contrast between citations received by the book [73] (about 900 citations) and the –identical– journal article [337] (some 2300 citations); the case illustrates the need to exert caution when dealing with different formats and vehicles of scientific communication, an issue that is getting ever more complex with digital formats and the Internet [11], [338]. Several other articles by Doll & Peto have each received over 1500 citations. The most cited paper by Willett [339] has received some 2000 citations, about half than his book [302]. But some sets of papers by him and other authors are, together, more cited than the reference book by the same author(s). The most cited papers by Cochrane [340], [341] received some 500 citations, less than half his book [69]. The top-cited paper by Rose [342] received some 1000 citations, similar to his book [233]; and again, several other papers of his have been highly cited. All articles coauthored by K. J. Rothman received over 12400 citations, a similar amount than *Modern epidemiology* [250]–[252]. Just two of several books by David G. Kleinbaum combined –*Applied regression analysis* [163]–[166] and *Epidemiologic research* [167]– received over 17000 citations (Table 2), whereas his articles have some 3000 citations.

To conclude, results may reflect a considerably positive influence of epidemiologic and statistical science on biomedical research [139], [140], [199]–[201]. On the other hand, results on the smaller number of citations received by theoretical and policy-oriented books cannot discern whether the influence was weaker on the ideas and policies that so much affect the health and wellbeing of citizens.

## Supporting Information

Appendix S1 **Details on Materials and Methods.**
(DOCX)

Table S1 Examples of books that received less than 25 citations.
(DOCX)

Figure S1 **Two examples of books whose titles were recorded in different ways in the Web of Science, the** ***Oxford Textbook of Public Health*** **and** ***Modern Epidemiology*** **.**
(TIFF)

Figure S2 **Citations to 4 books on causality (total, 2,529 citations).**
(TIF)

Figure S3 **Citations to 2 books by H. Blalock (1,705 citations).**
(TIF)

Figure S4 **Citations to 3 books on clinical trials: S.J. Pocock (2,421 citations), L.M. Friedman et al. (1,498 citations), and C. Meinert (703 citations).**
(TIF)

Figure S5 **Citations to 3 books on social epidemiology: Berkman & Kawachi (1,311 citations), Marmot & Wilkinson (1,214 citations) and Townsend & Davidson (1,364 citations).**
(TIF)

Figure S6 **Evolution over time of the 2,123 citations received by A. B. Hill's ‘Principles of medical statistics’.**
(TIF)

Figure S7 **Evolution over time of the 475 citations received by Susser's ‘Causal thinking in the health sciences’.**
(TIF)

Figure S8 **Evolution over time of the 733 citations received by 3 books by M. Khoury.**
(TIF)

Figure S9 **Citations to 2 books on occupational epidemiology: H. Checkoway et al. (619 citations) and R.R. Monson et al. (297 citations).**
(TIF)

Figure S10 **Trends of citations to the book by Hosmer & Lemeshow, and to the article by Mantel & Haenszel.**
(TIF)
