# Supplementary material for: Trends in Citations to Books on Epidemiological and Statistical Methods in the Biomedical Literature
Source: PLoS One. 2013 May 7;8(5):e61837. doi: 10.1371/journal.pone.0061837 (PMC3646840; doi:10.1371/journal.pone.0061837)
Supplement: Appendix S1 — Details on Materials and Methods. (DOCX) [file pone.0061837.s001.docx]

**Supplementary data.**

**1. Details on the search of citations**

In our searches, we used combinations of ‘cited author’ and ‘cited work’ as, for example, ‘(H*er AND Ap* log*’ for *Applied logistic regression* (by David W. Hosmer and Stanley Lemeshow). Such truncation gives results as ‘HASMER D APPL LOGISTIC REGRES - HOASMER DW – APPL LOGISTIC REGRES’, ‘HOEMER DW – APPL LOGISTIC REGRES’, ‘HOFMER DW – APPL LOGISTIC REGRES’, ‘HOSMER D – APPL LOG REGRESSION’. Books with more than one author or editor required searches for all authors or editors. In the previous example, we completed the search with ‘Lemes* AND Ap* log*’.

Errors in citations or in the transcription of citations from the articles to the data source abounded. For instance, the name of Fleiss (author of *Statistical methods for rates and proportions*) appears as Feiss, Feliss, Fieis, Fleis, Fless, Flies, Fliess, Leiss, Lfeiss, Mleiss, Reiss, Sleiss, Fkleiss, Fleisis or Fleise; and the title of the book (besides author ‘Fleiss’ or similar names), appears as ‘Stat Methods Weight P’, ‘Stat Methods Dates P’, ‘Stat Methods Goes Ra’ or Stat Meth Jbr Rat’. Such errors in citations require that searches are performed looking individually for many possible combinations. By looking at the actual citing articles we veryfied that sometimes the error in the citation appeared in the article, and sometimes in the data base.

Information on cited books in the data source was verified against valid information on all editions of the book; for instance, the year of publication that appeared in a citation in the data source was checked against information from the publisher’s web, from online catalogues of libraries (e.g., the British Library, Harvard’s Countway Library of Medicine and Hollis catalogues), from book pages available through search engines (e.g., Google Scholar) and websellers (e.g., Amazon).

Other examples of searches are:

*– Statistical methods* (Armitage et al.). Searched as ‘A*mi* AND st*’ it appears recorded as, for example, ‘AMITAGE – STAT METHODS MED RES’, ‘ARMI – STAT METHODS MED RES’, ‘ARMIAGE P – STATISTICAL METHODS’, ‘ARMIGTAGE PBG – STAT METHODS MED RES’, ‘ARMITAGE P – STA METHODS MED RES’. With this search we obtained some results of citations to articles published by the journal *Statistics in Medicine*, which we excluded, for instance, ‘ARMITAGE P – STAT MED – 1993 – [volume] 12 – [page] 1533’. We also searched the other editors of the book (e.g., as ‘Berry AND st* met*’ and ‘Matth* AND st* met*’).

*– Applied regression analysis* (Kleinbaum et al.). We searched for citations to ‘K* AND ap* reg*’. Thus, for instance, we got Cited Authors in results as ‘KEIBAUN DG – KELIMBAUM DG - KLEIBAUM DG’. As in previous examples we searched for all the editors of the book: ‘Kupp* AND ap* reg’, ‘Mull* AND ap* reg’, ‘Niz* AND ap* reg’.

– The book *Preventive medicine and hygiene* (Rosenau) raises another issue: it has changed title and editors in different editions; e.g.. Maxcy KF, ed., *Rosenau Preventive medicine and hygiene,* 7th. ed.; Sartwell PE, ed., *Maxcy-Rosenau Preventive medicine and public health,* 10th. ed.; Last JM, ed., *Maxcy-Rosenau Public health and preventive medicine,* 11th. ed.; Last JM, Wallace RB, eds., *Maxcy-Rosenau-Last Public health and preventive medicine,* 13th. ed; Wallace RB, ed. Maxcy-Rosenau-Last Public health and preventive medicine. 15th ed. New York: McGraw-Hill, 2008. We counted all citations to all editions taking into account all variants and possible errors mixing authors and titles. Thus, for instance, searched as ‘Rose* AND prev*’ we got results as ‘ROSENAN MJ – PREVENTIVE MED HYGIE’, ‘ROSENAU – PREVENTIVE MED HYGIE’ or ‘ROSENAU – PREVENTIVE MEDICINE – 1956’. The last result seems to be a citation to the second revision by Maxcy of Rosenau’s work, but the citing article actually considered Rosenau the only author. Further examples of the searches for this book are: ‘[Cited author] Ma*cy AND [Cited work] Rosen*’, ‘Ma*cy AND prev*’, ‘Last AND pub* h*’, ‘[Cited author] Last AND [Cited work] Maxcy*’, ‘[Cited author] Sar*w* AND [Cited work] Ma*cy*’, ‘Sartw* AND prev*’.

ISI - Thomson - Reuters data does not actually allow to reliably identify chapters of books written by authors other than the book editors, unless the title of the book is unique or unambiguous. The present study does include citations to chapters that were cited by the citing articles using the name of the book editor, the book title, and the pages corresponding to the chapter.

We next offer examples of books whose title was unambiguous and for which, therefore, the number of citations included in the present study includes all citations (i.e., it also includes citations to individual chapters not authored by the book editors):

– *Oxford Textbook of Public Health* (Holland et al.). When searched as ‘(oxf* t*x* p* h*) OR (oxf* t*x* pub* )’ and variants, it appears recorded in the data base as, for example, ‘OXFORD TXB PUBLIC HL’, ‘OXFORD TXB PULIC HLT, ‘ OXFORD TXB PUBL HLTH’ ‘OXFORD TEXTBOOK PUBL’ See also Supplementary data, Figure 1.

– *Modern Epidemiology* (Rothman et al.). When searched as ‘mod* epi*’, it appears recorded in the data base as, for example, ‘MODERN EPIDEMIOLOGY’. The truncation ‘mod* epi*’ required us to discard one by one citations to books registered in the data base as, for instance, ‘MODELING AIDS EPIDEM’, ‘MODELLING EPISODES A’ or ‘MODELS SEIZURES EPIL’. Citations to articles whose titles begin with words similar to the initial title of the book of interest were also easily excluded during this phase; in the ISI - Thomson - Reuters data base it is easy to differentiate a citation to an article and a citation to a book. A longer truncation as, for instance, ‘modern epi*’ would miss citations with mistakes as ‘MODEN EPIDEMIOLOGY’.

– *Teaching epidemiology* (Olsen et al.). Searched as ‘tea* epi*’, it appears recorded in the data base as, for example, ‘OLSEN J – TEACHING EPIDEMIOLOG – 1992’ or as ‘FLOREY CD – TEACHING EPIDEMIOLOG – 1992 – [page] 23’. When a citation mistake was obvious, the citation was counted (e.g., we did include one citation to ‘OLSEN J – TEACHING EPIDEMIOLOG – 1993’, instead of 1992). By checking each possible citation there was little doubt that certain citations should not be counted (e.g., ‘ROLLESTON TW – TEACHING EPICTETUS B – 1888’ or ‘VOS G – TEACHING EPIST HEBRE – 1956).

For the following books it was feasible to search and count citations to individual chapters, either because the title was unequivocal or because we specifically searched for citations that included the name of the first author of each and all chapters plus the title of the book; we also started by identifying all citations that included the name of the book, looked at the names of the authors of such citations, verified which names where actually authors of chapters of the book, and counted such citations as citations to the book (please, see full citations in references of main text):

– Rothman KJ. *Modern epidemiology* (1986). Rothman KJ, Greenland S, eds. *Modern epidemiology*. 2nd. ed. (1998). Rothman KJ, Greenland S, Lash TL, eds. *Modern epidemiology*. 3rd. ed. (2008).

– Holland WW, Detels R, Knox, eds *Oxford Textbook of Public Health* (1984) and 2nd ed. (1991). Detels R, McEwen J, Beaglehole T, Tanaka H, eds *Oxford Textbook of Public Health* 3rd ed.(1997) and 4th ed. (2002).Detels R, Beaglehole T, Lansang MA, Gulliford M, eds. *Oxford Textbook of Public Health*. 5th. ed. (2009).

– Jensen O, Parkin DM, MacLennan R, Muir CS, Skeet R. *Cancer registration* (1991).

– Adami HO, Hunter D, Trichopoulos D, eds. *Textbook of cancer epidemiology*. 1st. ed. (2002),

2nd. ed. (2008).

– Nelson KE, Williams CM, Graham NMH. *Infectious diseases epidemiology. Theory and practice*. 1st. ed. (2001) and 2nd. ed. (2007).

– Khoury MJ, Little J, Burke W. *Human genome epidemiology* 1st ed. (2003). Khoury MJ, Bedrosian S, Gwinn M, et al., eds *Human genome epidemiology* 2nd ed. (2010).

– Gregg MB, ed. *Field epidemiology*. 1st. ed. (1996), 2nd ed. (2002), 3rd ed. (2008).

– Olsen J, Trichopoulos D, eds. *Teaching epidemiology*. 1st. ed. (1992). Olsen J, Saracci R, Trichopoulos D, eds. *Teaching epidemiology*. 2nd. ed. (2001), 3rd ed. (2010).

– Buck C, Llopis A, Nájera E, Terris M, eds. *The challenge of epidemiology* (1988).

– Elston R, Olson J, Palmer L, eds. *Biostatistical genetics and genetic epidemiology* (2002).

– Toniolo P, Boffetta P, Shuker DEG, et al., eds. *Application of biomarkers in cancer epidemiology* (1997).

– Armenian HK, Shapiro S. *Epidemiology and health services* (1998).

– Ashton J, ed. *The epidemiological imagination: A reader* (1994).

– Wild C, Vineis P, Garte S, eds. *Molecular epidemiology of chronic diseases* (2008).

– Carrington M, Hoelzel R. *Molecular epidemiology* (2001).

– Rebbeck TR, Ambrosone CB, Shields PG, eds. *Molecular epidemiology: applications in cancer and other human diseases* (2008).

– Hartzema AG, Porta MS, Tilson HH, eds*. Pharmacoepidemiology. An introduction*. 2nd ed (1991), 3rd ed (1998). Hartzema AG, Tilson HH, Chan KA, eds *Pharmacoepidemiology and therapeutic risk management* (2008). In this case we did as explained at the start of this paragraph, additionally taking into account that several authors of chapters had written chapters in the book edited by Brian Strom (*Pharmacoepidemiology*); it was possible to distinguish between the two books through the year of publication, by reading the reference in the citing article, through the Digital object identifier (doi), PubMed or the citing journal’s web page. In addition, several citations cited articles published by authors of Hartzema’s et al. book in the journal *Pharmacoepidemiology and drug safety*, which it was feasible to identify (and not count as citations to the book) since such citations included as source ‘PHARMACOEPIDEM DR S’, ‘PHARMACOEPIDEMIOL S1’ and variants.

By contrast, the title of some books is unspecific or similar to a journal’s name; hence, it was not possible to identify all citations using the name of the first author of chapters not authored by the book editors. Examples include:

– Schottenfeld D, Fraumeni FJ Jr, eds. *Cancer epidemiology and prevention* (1st. ed.: 1982).

– Rosenau, Maxcy, Sartwell, Last, eds. *Preventive medicine* (1st. ed.: 1913).

– Hulley SB, Cummings SR, eds. *Designing clinical research* (1st. ed.: 1988).

– Strom BL, ed. *Pharmacoepidemiology* (1st. ed.: 1989).

– Sackett DL et al, ed. *Evidence-based medicine: how to practice and teach EBM* (1st ed.: 1997). The title of this book partly coincides with the name of the journal *Evidence-based medicine for primary care and internal medicine*. To distinguish between citations to the book and to articles in the journal by the book authors we looked at the references in the citing articles; again, the articles were identified through the Digital object identifier (doi) or PubMed. It was often possible to distinguish between citations to the book and to articles in the journal because the year of publication in the citation was not one of the years of publication of any of the editions of the book, or because the citation included an article ID, although none of these two circumstances was required to make the distinction. Citations that started as ‘EVIDENCE BASED MED H’ were safely attributable to the book. Citations to works in “EVIDENCE BASED MED” could be to the book or the journal articles, and were checked as mentioned; most were citations to the book. Citations to other books presenting similar issues were treated in the same way.

Certainly, titles of some books may be thought to fall within the two groups mentioned above (i.e., books with unambiguous and common titles); for instance:

– Jensen O, Parkin DM, MacLennan R, Muir CS, Skeet R. *Cancer registration: principles and methods*. Lyon: International Agency for Research on Cancer, 1991. We searched for it with ‘canc* reg*’ OR ‘princi* meth*’and checked the year of publication (as well as the authors, of course). When we used ‘canc* reg* p*’, 9 citations were missed, including 8 to ‘JENSEN OM – CANCER REGISTRATION – 1991’. As mentioned above, it was feasible to search and count citations to individual chapters.

Additional examples of books that could not be assessed for inclusion are:

– IARC Monographs on the Evaluation of Carcinogenic Risks to Humans, IARC Handbooks of Cancer Prevention, and the series of *Cancer Incidence in Five Continents*.

– White KL, Frenk J, Ordóñez C, et al., eds. *Health services research: An anthology*. Washington, DC: Pan American Health Organization; 1992.

In summary, if the title was unambiguous, the number of citations included in the present study includes citations to individual chapters not authored by the book editors; if the title of the book was common, we tried to include all citations registered with the name of the first author of each chapter, as explained above.

At present, the data source does not permit to include citations to books made in other books.

**2. Referents: books not focused on epidemiology and biostatistics**

We next provide a few examples of texts (mostly, on health policy, philosophy of medicine and related areas) in which epidemiology and biostatistics have a marginal role, but which nevertheless may act as referents of the study main findings:

– Paul Starr. The social transformation of American medicine (1st. ed., 1982): 3,121 citations.

– Ivan Illich. Medical nemesis (1st. ed.; 1975); Limits to medicine: Medical nemesis (2nd. ed.: 1976) (1977-1982): 2,024 citations.

– Arthur Kleinman. The illness narratives. Suffering, healing and the human condition (1st. ed., 1988): 1,640 citations.

– Victor Fuchs. Who shall live? Health, economics, and social choice (editions of 1974, 1975, 1983, 1986, 2001): 628 citations.

– John H. Knowles. Doing better and feeling worse. Health in the United States (1st. ed., 1977): 325 citations (including citations to chapters).

– McMichael AJ. Human frontiers, environments and diseases. Past patterns, uncertain futures. Cambridge: Cambridge University Press (1st. ed., 2001): 154 citations.

– Siegrist J, Marmot M. Social inequalities in health. New York: Oxford University Press (1st. ed., 2006): 140 citations (including citations to chapters).

– Kawachi I, Kennedy BP. The health of nations. Why inequality is harmful to your health. New York: The New York Press (1st. ed., 2002): 94 citations (including citations to chapters).

– Kawachi I, Wamala S. Globalization and health. New York: Oxford University Press (1st. ed., 2006): 22 citations.

– Milos Jenicek. Foundations of evidence-based medicine (1st. ed., 2003): 34 citations.

– Jenicek M, Hitchcock DL. Evicence-based practice. Logic and critical thinking in medicine. Chicago: American Medical Association (AMA Press) (1st. ed., 2005): 20 citations.

– Jenicek M. A physician's self-paced guide to critical thinking. Chicago: American Medical Association (AMA Press) (1st. ed., 2006): 9 citations.

**3. Books not included in the study**

If a book is not included in this article (e.g., it does not appear in Table 2) in spite of appearing to fulfill inclusion criteria, chances are that the number of citations it received was less than 25. Examples of such books are given in Supplementary Table S1.
