# Supplementary material for: Trends in Citations to Books on Epidemiological and Statistical Methods in the Biomedical Literature
Source: PLoS One. 2013 May 7;8(5):e61837. doi: 10.1371/journal.pone.0061837 (PMC3646840; doi:10.1371/journal.pone.0061837)
Supplement: Table S1 — Examples of books that received less than 25 citations. (DOCX) [file pone.0061837.s002.docx]

**Supplementary data.**

**Supplementary Table S1. Examples of books that received less than 25 citations.***

| **Author(s)** | **Brief title** | **Edition,**  **year** | **Years** | **Citations**  **(total)** | Citations  per year | |
| --- | --- | --- | --- | --- | --- | --- |
| Blalock | Causal models in panel and experimental designs | 1st., 1985 | 26 | 23 | 0.9 |  |
| Wild, Vineis, Garte | Molecular epidemiology | 1st., 2008 | 3 | 22 | 7.3 |  |
| Prince, et al. | Practical psychiatric epidemiology | 1st., 2003 | 8 | 22 | 2.8 |  |
| Page, Cole, Timmreck | Basic epidemiological methods | 1st., 1995 | 16 | 22 | 1.4 |  |
| Roberts | Epidemiology for clinicians | 1st., 1977 | 34 | 21 | 0.6 |  |
| Norell | Workbook of epidemiology | 1st., 1995 | 16 | 18 | 1.1 |  |
| Ashton | Epidemiological imagination | 1st., 1994 | 17 | 12 | 0.7 |  |
| Rebbeck, Ambrosone, Shields | Molecular epidemiology | 1st., 2008 | 3 | 9 | 3.0 |  |
| Unwin, Carr, Leeson | Introductory study guide | 1st., 1997 | 14 | 9 | 0.6 |  |
| Sydenstricker | Health and environment | 1st., 1933  2nd., 1972 | 78 | 9 | 0.1 |  |
| Hudson, et al. | Clinical preventive medicine | 1st., 1988 | 23 | 8 | 0.3 |  |
| Schuman | Practice-based epidemiology | 1st., 1986 | 25 | 8 | 0.3 |  |
| Morrell | Epidemiology in general practice | 1st., 1988 | 23 | 6 | 0.3 |  |
| Carrington, Hoelzel | Molecular epidemiology | 1st., 2001 | 10 | 5 | 0.5 |  |
| Harkness | Epidemiology in nursing practice | 1st., 1995 | 16 | 3 | 0.2 |  |
| Steenland | Case studies in occupational epidemiology | 1st., 1993 | 18 | 2 | 0.1 |  |
| Gray, Fowler | Essentials of preventive medicine | 1st., 1984 | 27 | 2 | 0.1 |  |
| Waning, Montagne | Pharmacoepidemiology | 1st., 2000 | 11 | 1 | 0.1 |  |
| Loue | Case studies in forensic epidemiology | 1st., 2002 | 9 | 1 | 0.1 |  |
| Winklestein, French, Lane | Basic readings in epidemiology | 1st., 1970 | 41 | 1 | 0.0 |  |

*Please see book references, and footnotes to Table 2 in the article’s main text.
