# Supplementary figures and images for: Trends in Citations to Books on Epidemiological and Statistical Methods in the Biomedical Literature
Source: PLoS One. 2013 May 7;8(5):e61837. doi: 10.1371/journal.pone.0061837 (PMC3646840; doi:10.1371/journal.pone.0061837)

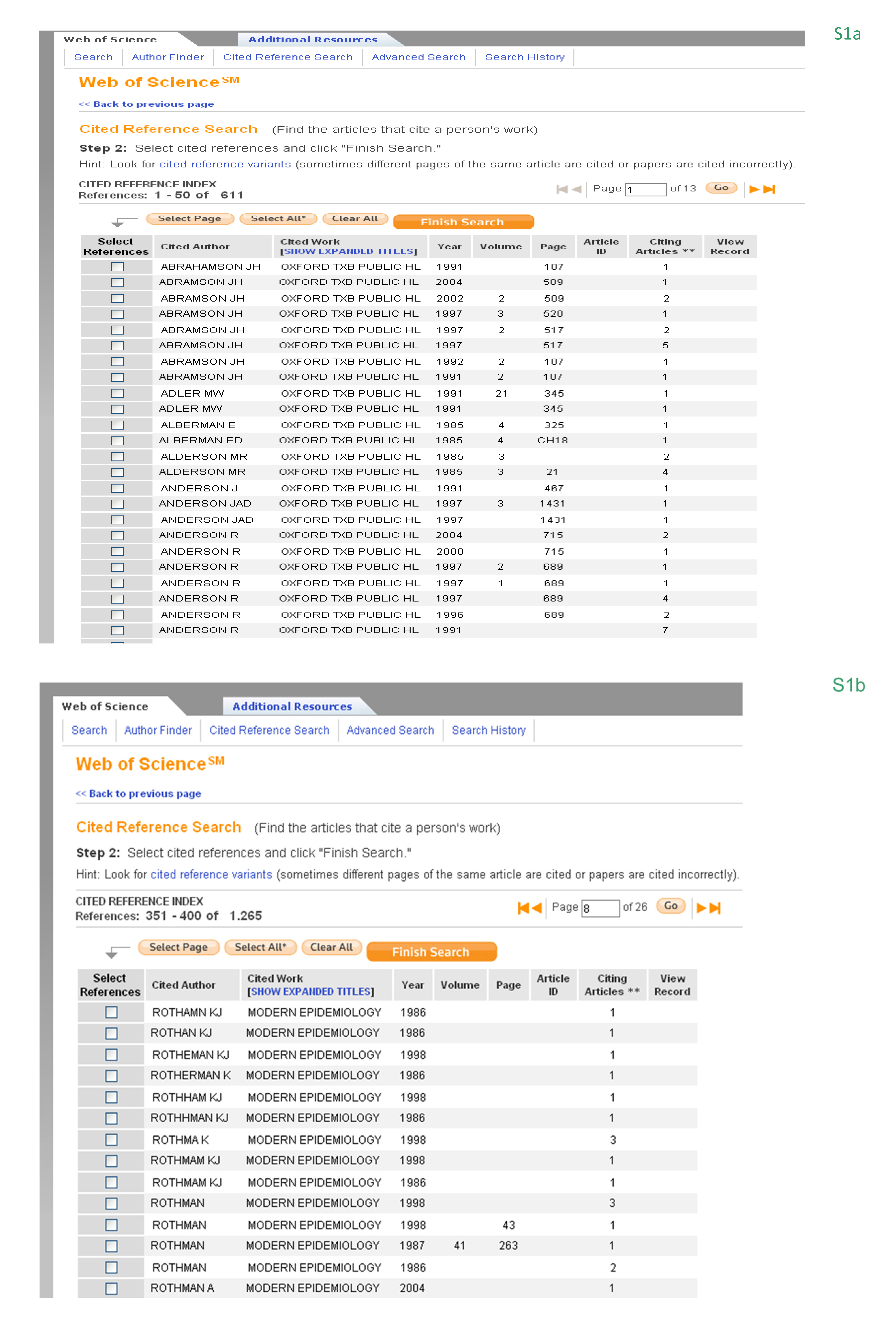

Supplement: Figure S1 — Two examples of books whose titles were recorded in different ways in the Web of Science, the Oxford Textbook of Public Health and Modern Epidemiology . (TIFF) [file pone.0061837.s003.tif]

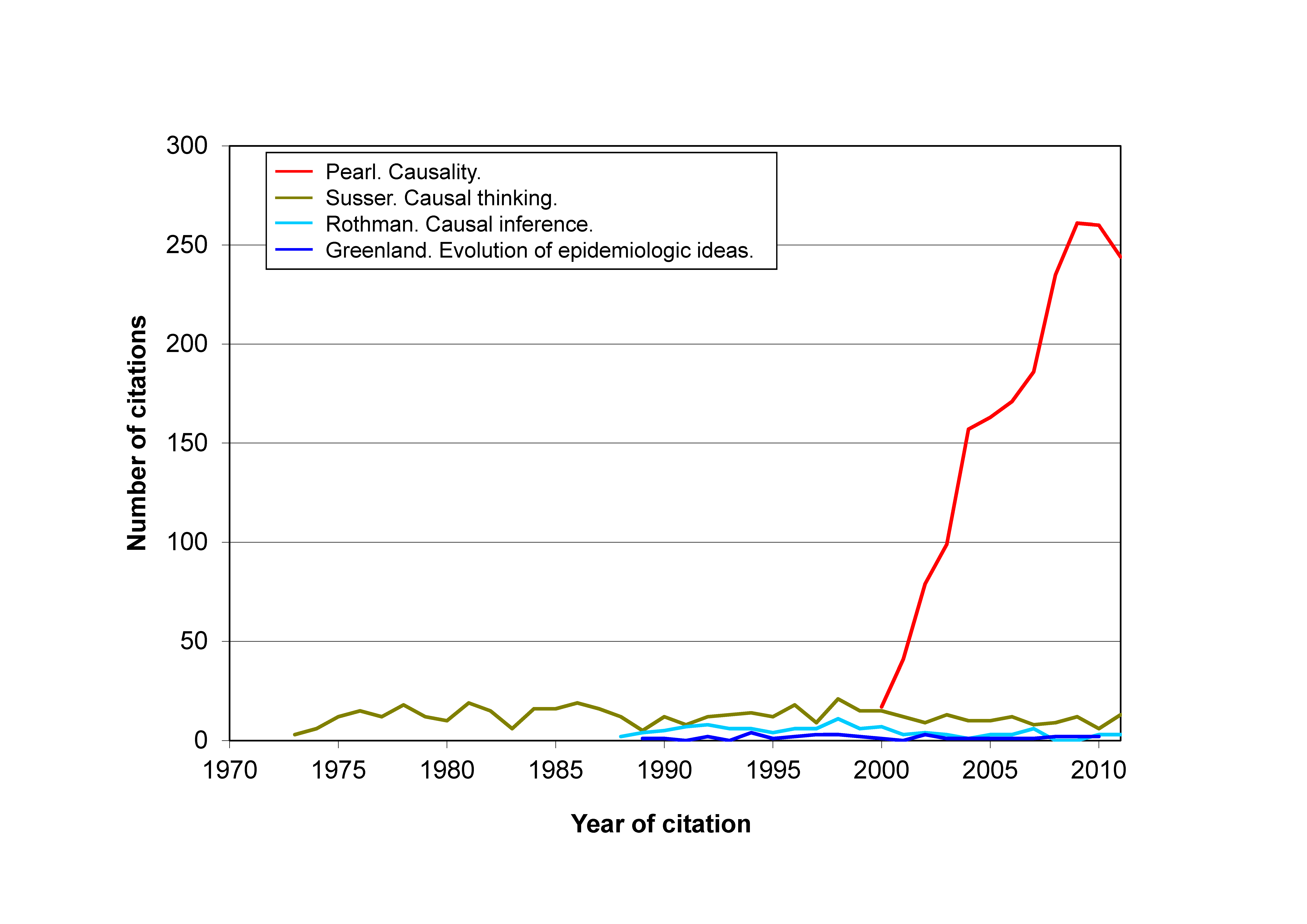

Supplement: Figure S2 — Citations to 4 books on causality (total, 2,529 citations). (TIF) [file pone.0061837.s004.tif]

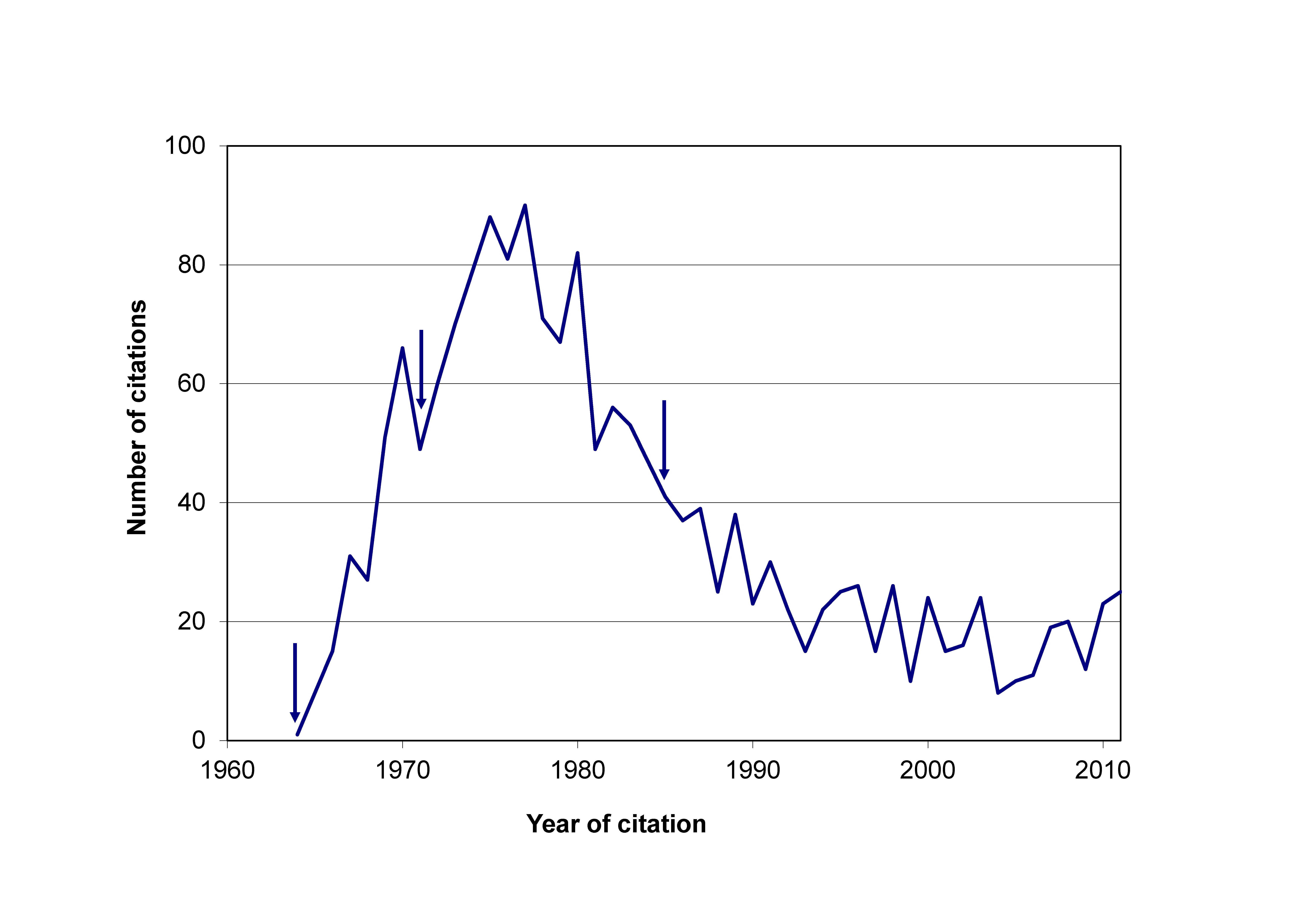

Supplement: Figure S3 — Citations to 2 books by H. Blalock (1,705 citations). (TIF) [file pone.0061837.s005.tif]

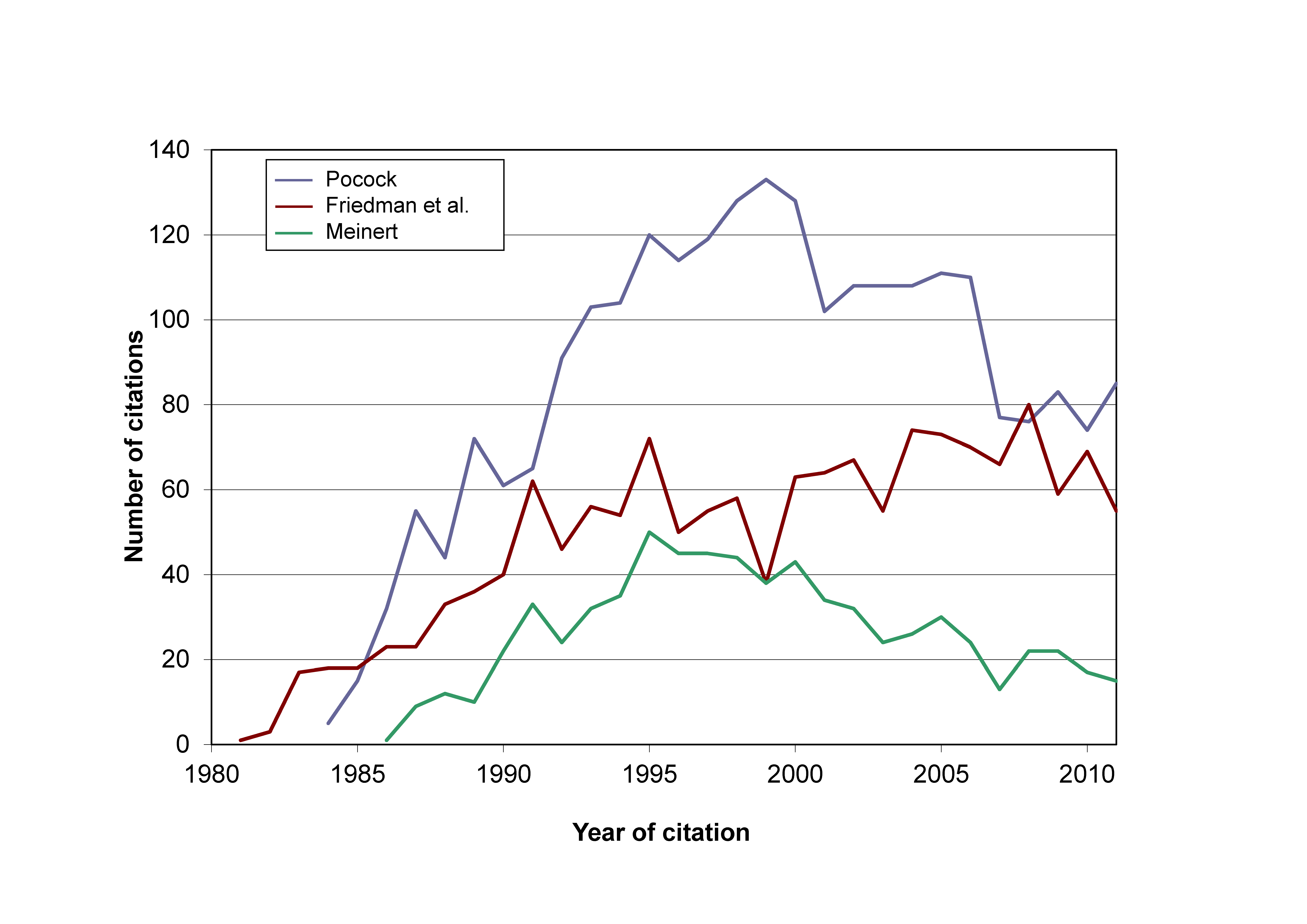

Supplement: Figure S4 — Citations to 3 books on clinical trials: S.J. Pocock (2,421 citations), L.M. Friedman et al. (1,498 citations), and C. Meinert (703 citations). (TIF) [file pone.0061837.s006.tif]

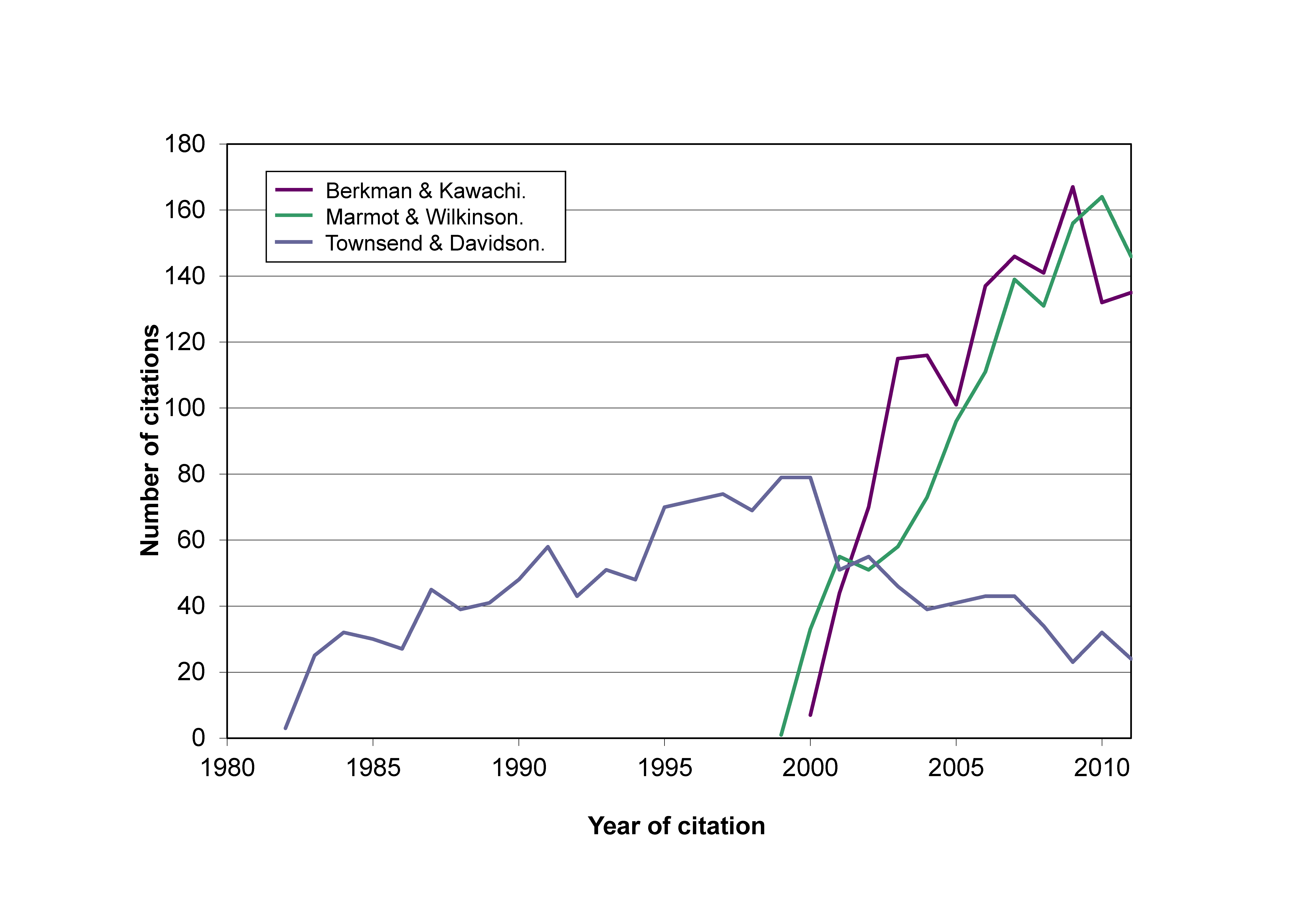

Supplement: Figure S5 — Citations to 3 books on social epidemiology: Berkman & Kawachi (1,311 citations), Marmot & Wilkinson (1,214 citations) and Townsend & Davidson (1,364 citations). (TIF) [file pone.0061837.s007.tif]

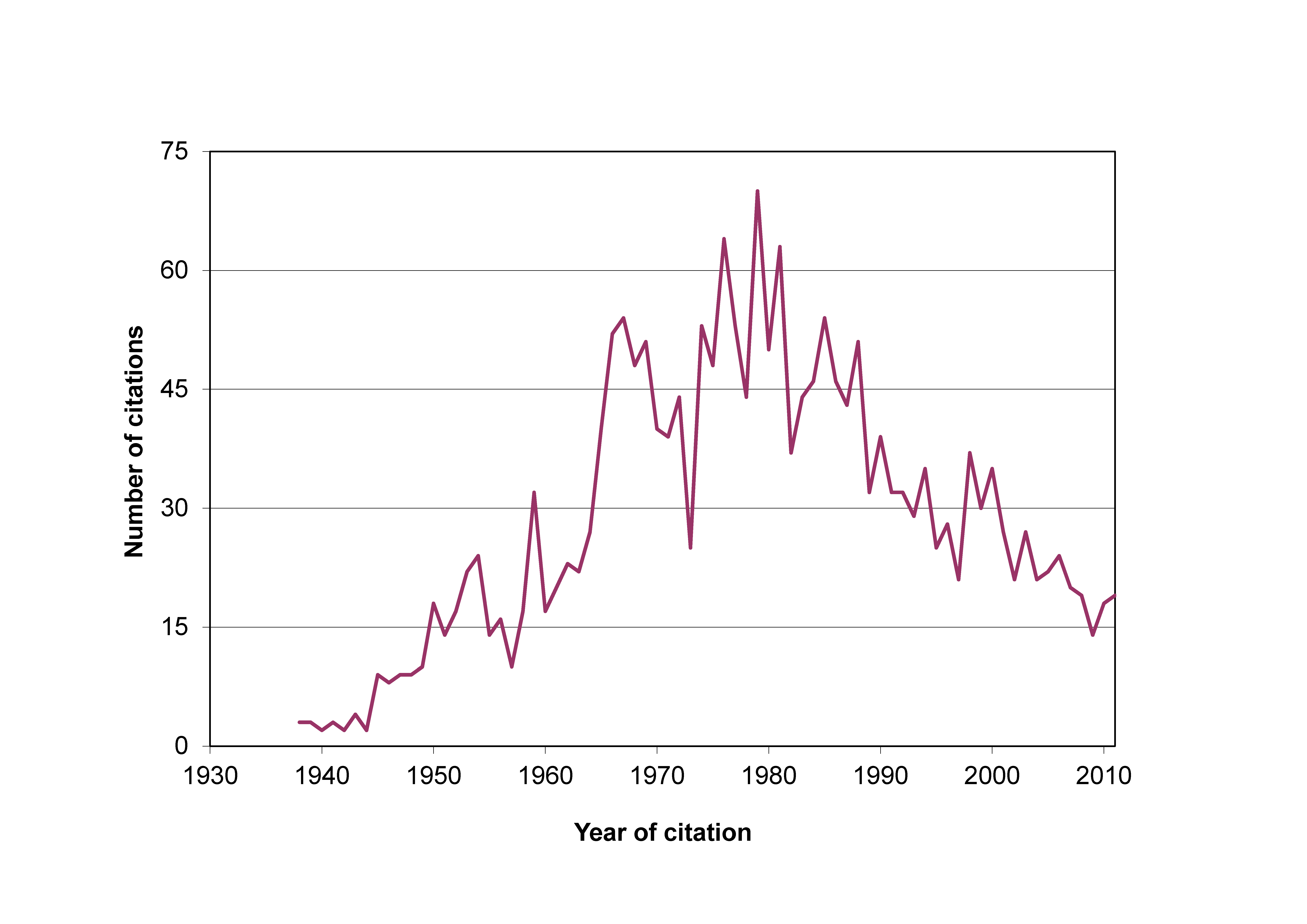

Supplement: Figure S6 — Evolution over time of the 2,123 citations received by A. B. Hill's ‘Principles of medical statistics’. (TIF) [file pone.0061837.s008.tif]

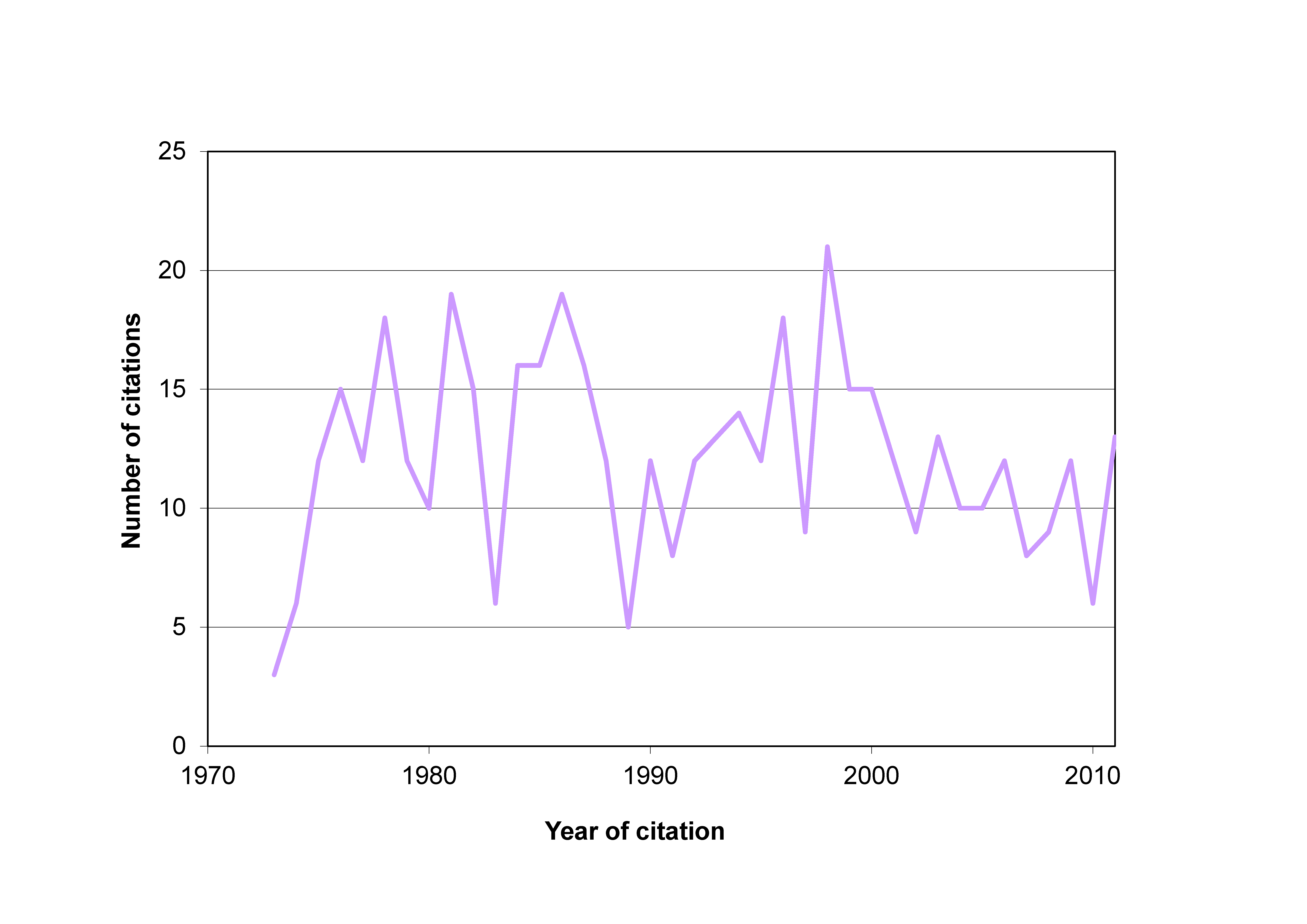

Supplement: Figure S7 — Evolution over time of the 475 citations received by Susser's ‘Causal thinking in the health sciences’. (TIF) [file pone.0061837.s009.tif]

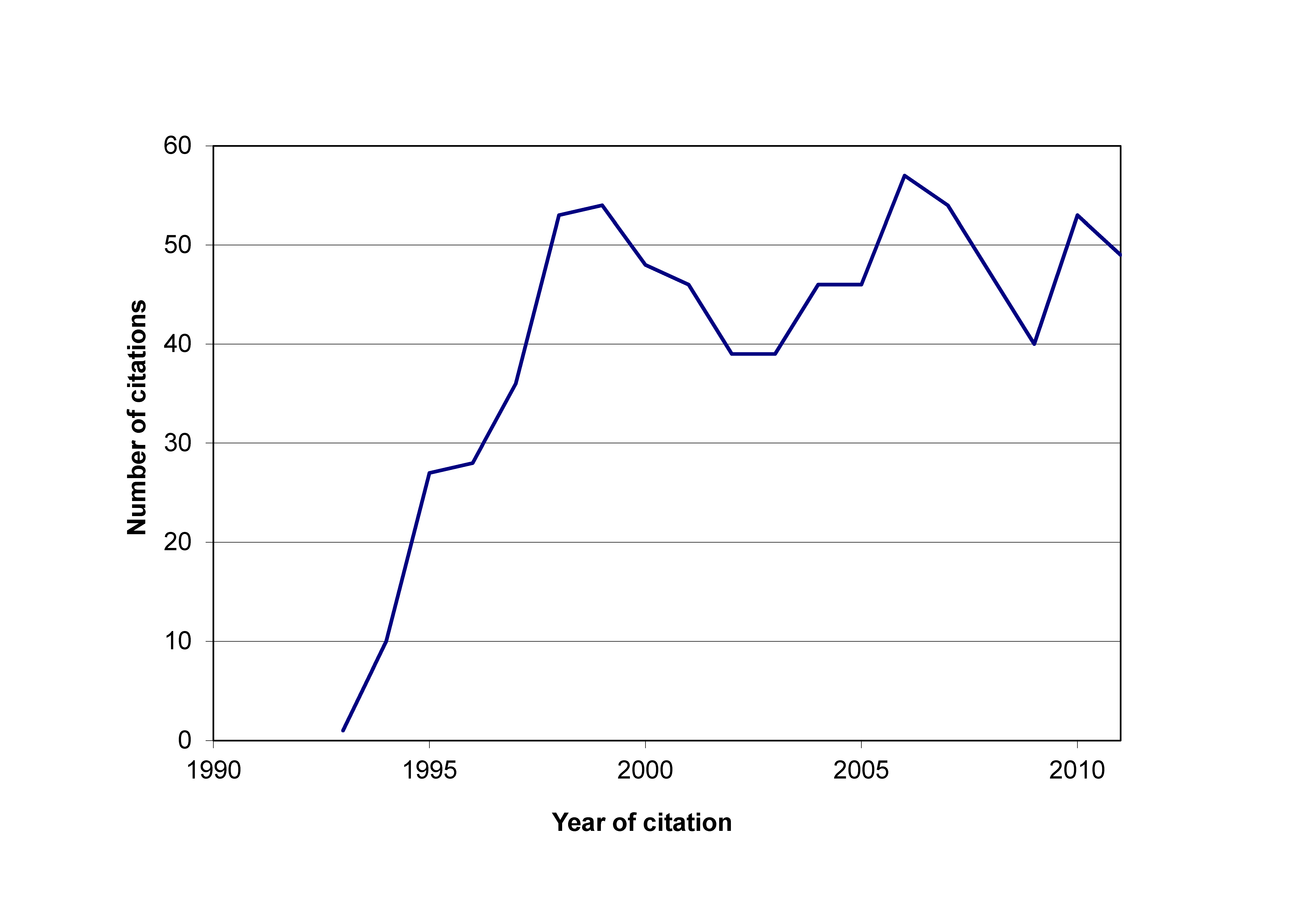

Supplement: Figure S8 — Evolution over time of the 733 citations received by 3 books by M. Khoury. (TIF) [file pone.0061837.s010.tif]

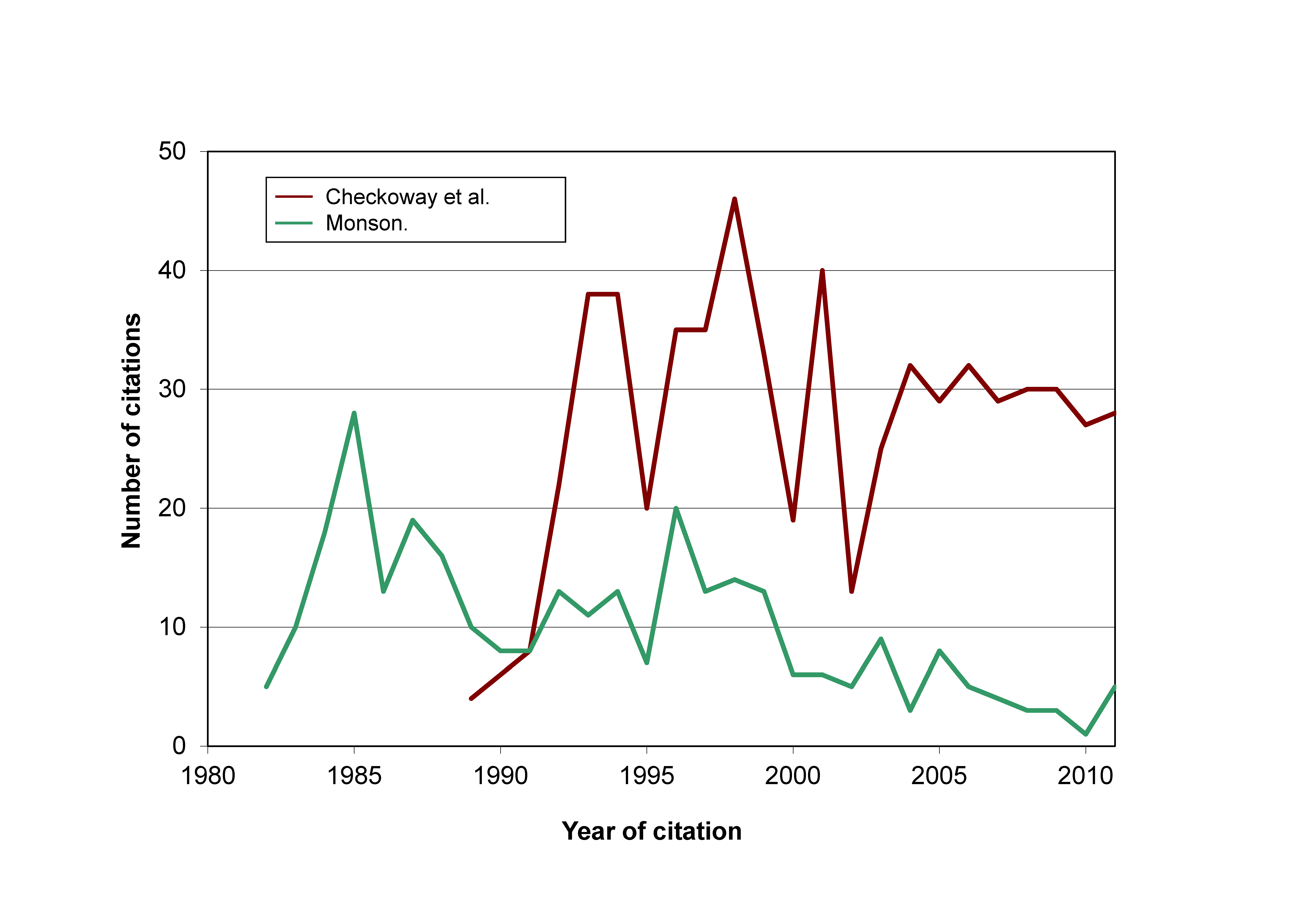

Supplement: Figure S9 — Citations to 2 books on occupational epidemiology: H. Checkoway et al. (619 citations) and R.R. Monson et al. (297 citations). (TIF) [file pone.0061837.s011.tif]

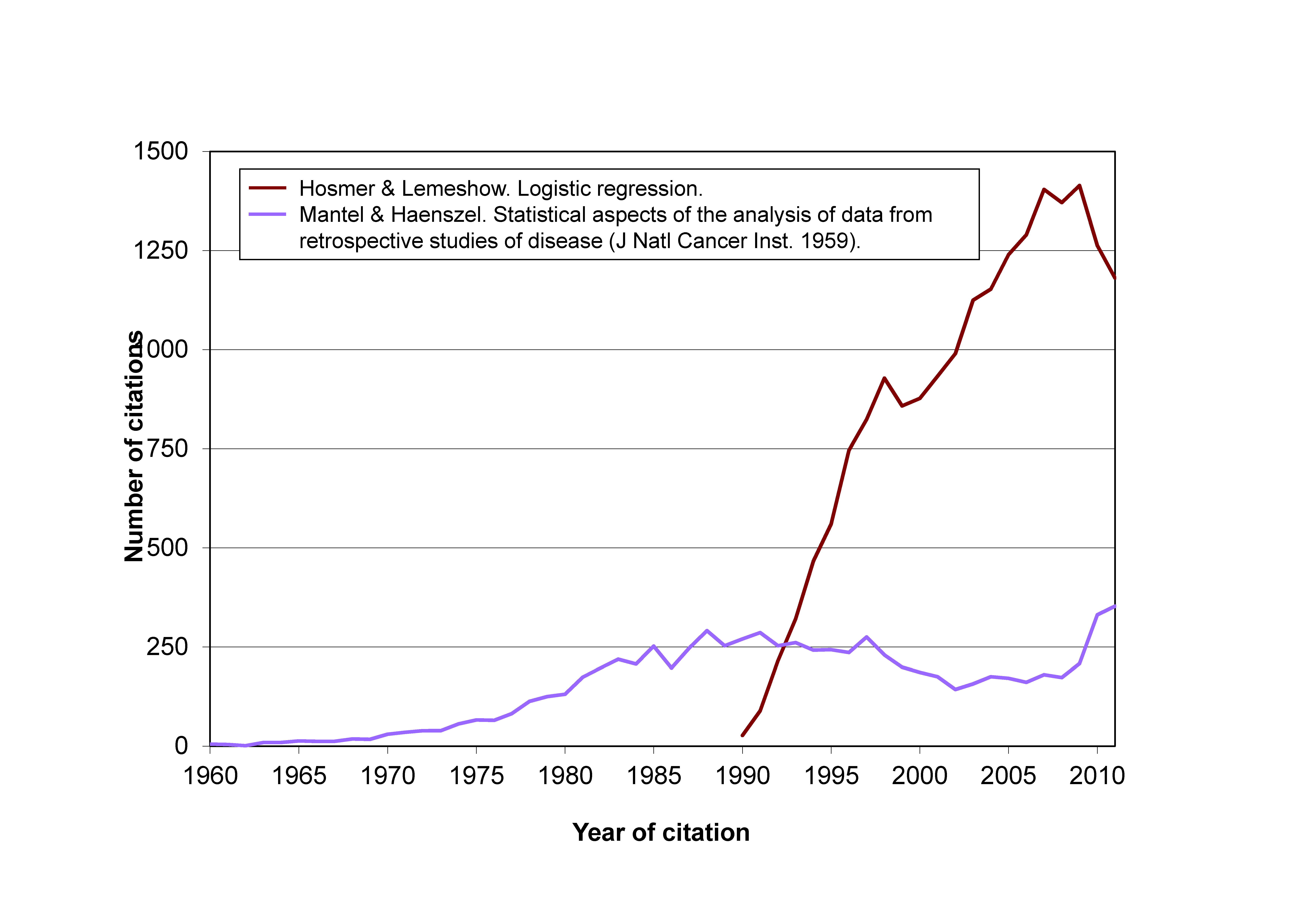

Supplement: Figure S10 — Trends of citations to the book by Hosmer & Lemeshow, and to the article by Mantel & Haenszel. (TIF) [file pone.0061837.s012.tif]
